# Modeling the two- and three-dimensional displacement field in Lorca, Spain, subsidence and the global implications

**DOI:** 10.1038/s41598-018-33128-0

**Published:** 2018-10-03

**Authors:** Jose Fernandez, Juan F. Prieto, Joaquin Escayo, Antonio G. Camacho, Francisco Luzón, Kristy F. Tiampo, Mimmo Palano, Tamara Abajo, Enrique Pérez, Jesus Velasco, Tomas Herrero, Guadalupe Bru, Iñigo Molina, Juan López, Gema Rodríguez-Velasco, Israel Gómez, Jordi J. Mallorquí

**Affiliations:** 1 Instituto de Geociencias (CSIC, UCM), Calle del Doctor Severo Ochoa, no 7, Facultad de Medicina (Edificio Entrepabellones 7 y 8, 4a planta), Ciudad Universitaria, 28040 Madrid, Spain; 2 ETSI Topografía, Geodesia y Cartografía, Universidad Politécnica de Madrid, Ctra. Valencia km 7, 28031 Madrid, Spain; 3 Dpto de Química y Física, Universidad de Almería, Edificio CITE-IIA, Cañada de San Urbano s/n, 04120 Almería, Spain; 4 Cooperative Institute for Research in Environmental Sciences (CIRES), 216UCB, University of Colorado at Boulder, Boulder, CO 80309 USA; 5 Istituto Nazionale di Geofisica e Vulcanologia, Osservatorio Etneo, 95125 Catania, Italy; 6 Dpto. Ingeniería Agroforestal, ETSI Agronómica, Alimentaria y de Biosistemas, Universidad Politécnica de Madrid, Avda. Puerta de Hierro, no 2 – 4, 28040 Madrid, Spain; 7 Dpto. Física de la Tierra y Astrofísica, Unidad Departamental Astronomía y Geodesia, Universidad Complutense de Madrid, Fac. C. Matemáticas, Plaza de Ciencias, 3, 28040 Madrid, Spain; 8 CommSensLab, Dep. Signal Theory and Communications, Universitat Politècnica de Catalunya (UPC), D3-Campus Nord-UPC, C. Jordi Girona 1-3, 08034 Barcelona, Spain

## Abstract

Land subsidence associated with overexploitation of aquifers is a hazard that commonly affects large areas worldwide. The Lorca area, located in southeast Spain, has undergone one of the highest subsidence rates in Europe as a direct consequence of long-term aquifer exploitation. Previous studies carried out on the region assumed that the ground deformation retrieved from satellite radar interferometry corresponds only to vertical displacement. Here we report, for the first time, the two- and three-dimensional displacement field over the study area using synthetic aperture radar (SAR) data from Sentinel-1A images and Global Navigation Satellite System (GNSS) observations. By modeling this displacement, we provide new insights on the spatial and temporal evolution of the subsidence processes and on the main governing mechanisms. Additionally, we also demonstrate the importance of knowing both the vertical and horizontal components of the displacement to properly characterize similar hazards. Based on these results, we propose some general guidelines for the sustainable management and monitoring of land subsidence related to anthropogenic activities.

## Introduction

Land subsidence, ranging from local collapse to the broad regional lowering of Earth’s surface, represents the main geomechanical effect related to the removal of subsurface support. Subsidence can occur as a result of (i) natural factors (e.g., tectonic activity, self-consolidation of recent sedimentary deposits, oxidation and shrinkage of organic soils)^1,2^ and (ii) anthropogenic processes (e.g., groundwater pumping^3–6^, urban development^7^, hydrocarbon or mining exploitation^8,9^).

In this study, we focus on land subsidence related to groundwater pumping because it represents a hazard commonly affecting large areas worldwide, usually associated with the increasing demand upon groundwater resources due to expanding metropolitan and agricultural areas in semiarid and arid regions^4^. The surface ground deformation thus constitutes a signature of the processes in the reservoir and can provide information about those subsurface processes^10^. A number of recent studies have focused on this topic^3–5,11–22^.

Frequently, land settlement goes unnoticed, only to be discovered later, after severe damage has occurred or in the framework of broader scientific or technical studies^4,7,12^. Recently, awareness on the damage threat posed by anthropogenic subsidence has increased significantly at both the political and public levels, thus contributing to lowering of the alarm threshold^12^. As a result, recent plans for subsurface resource management, including the study of the related environmental impact, have incorporated numerical predictions of the anticipated subsidence in the specific area of interest. Also in this context, the issue of anthropogenic land subsidence was included as one of the most urgent threats to sustainable development in the UNESCO International Hydrological Program VIII (2014–2020)^12,23^.

The modelling of surface deformation patterns can provide significant insights into the temporal changes of pore pressure as well as the 3D geometry of a reservoir in response to its exploitation over the time^16,21^. A number of different techniques have been developed in recent decades to estimate the surface deformation pattern related to volume changes in elastic and poroelastic media^6,21,24–29^. Inverse modeling is required to achieve success in such an endeavor^10,30^. A proper understanding of the subsidence mechanism is essential to calibrate protocols and best practices for monitoring natural and anthropogenic phenomena, with the aim to reduce vulnerability and risk for infrastructures, economies, natural environments and human life.

Given the limitations on the type and/or number of observation data, and on the geophysical and geological information for the study area, analytical models are used to estimate the amplitude and pattern of surface deformation based on assumptions about the media and perturbation source (e.g., using elastic or poroelastic theory)^29,31,32^. They provide a relatively simple method to model surface deformation for reservoirs of any geometric shape. Furthermore, given that these techniques assume that most of the surface deformation is explained by the poroelastic expansion or contraction of the reservoir, less in situ geological data is required than that needed for numerical models^29^.

One method of computing surface deformation is Geerstma’s nucleus of strain model in a half space^24,25^, in which pressure change occurs within many small prisms in the reservoir. Surface deformation can be computed by adding the influence of these depleting prisms. Given that Geertsma’s models are linear and the entire subsurface is assumed to be isotropic, superposition is allowable. Using this assumption, these linear equations permit the computation of surface deformation based on the superposition of many prismatic blocks within a compacting reservoir of any geometric shape^24^. See the Methods section for more details on the forward model and on the inversion technique^30,32,33^ used in this work.

**Figure 1:**
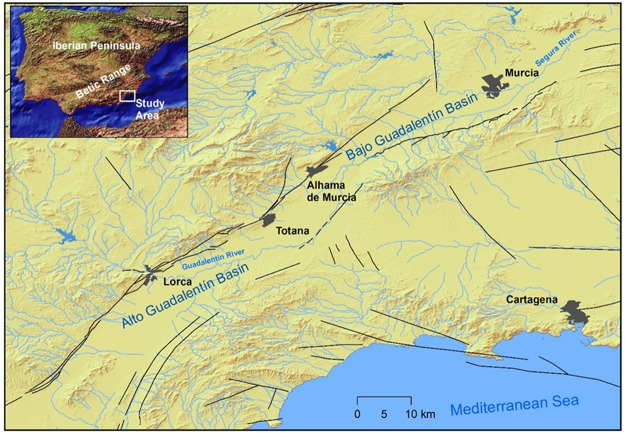
Geographical location of the study area. Location of the Alto Guadalentín Basin, the Bajo Guadalentín Basin and the Guadalentín River that formed the two basins. Black lines depict main faults in the area. The locations and names of the main cities in the area are shown. The topography has been obtained from MDT05 2015 CC-BY 4.0 digital elevation model^74^. This figure was generated using Arc Map 10.3 (http://desktop.argis.com/es/arcmap/).

**Figure 2:**
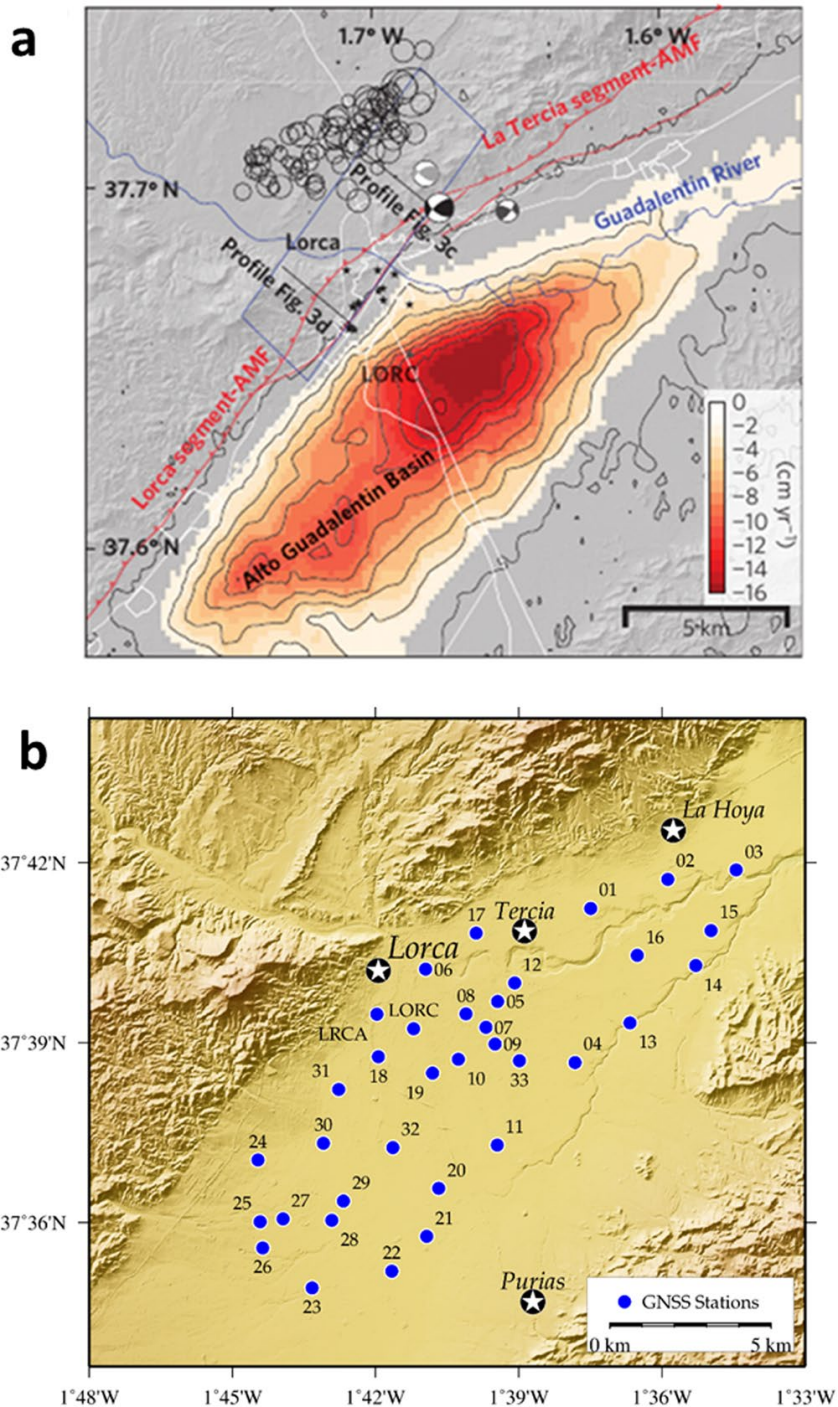
Subsidence area and location of the GNSS stations. (**a**) Subsidence area detected in previous studies^31^ by means of InSAR techniques along the Alto Guadalentín Basin. Subsidence rates have a maximum of 16 cm/yr for the period 2006–2011 located ~4 km south-west the city of Lorca. The black stars are damage locations due to the M = 5.1 May 2008 Lorca earthquake. Red lines are main faults (AMF, Alhama de Murcia Fault). The contour lines indicate 2 cm/yr InSAR subsidence due to groundwater pumping. (**b**) Location of the monitoring GNSS control stations deployed in the area of Alto Guadalentín. The network consists of 33 monitoring stations (blue circles show their location) and covers an area of about 70 km^2^. The network is designed to allow high accuracy GNSS surveys and also includes two existing continuous GNSS stations. Main population centers are depicted with white stars. GMT software was used to create this figure^75^.

The Lorca region, located in the Alto Guadalentín Basin of southeastern Spain (Fig. 1), is affected by subsidence rates of up to 10 cm/yr as a direct consequence of long-term aquifer exploitation^4,5^ (Fig. 2). This region is characterized by semi-arid climate conditions, with average precipitation rates of 150 mm/yr and an average annual temperature^34^ of ~18 °C. The basin is infilled with Quaternary alluvial fan systems overlapping Tertiary sediments transported by the Guadalentín River along the depression located in the eastern part of the Betic Mountain Range (an ENE-WSW oriented alpine orogenic belt resulting from the Nubia-Iberia ongoing convergence^35–37^).

The Guadalentín Basin aquifer is composed of two contiguous sub-basins: Alto and Bajo Guadalentín (Fig. 1). From a hydrogeological point of view, the basement beneath the aquifer is composed of several relatively impermeable Paleozoic metamorphic complexes overlain by permeable Miocene conglomerate and/or calcarenite series. The top of the succession comprises Pliocene-Quaternary, low-permeability, compressible conglomerates, sand, silt, and clays^4,38^. The Alto Guadalentín aquifer covers an area of approximately 277 km^2^. Historically, piezometric levels were located closely to the land surface, allowing the development of a number of artesian wells and permanent lagoons^38^. Since the 1960s–1970s, the Guadalentín Basin aquifer has reflected gradually increased overdraft and contamination (e.g., high electrical conductivity, CO_2_ positive thermal anomaly), and was legally declared provisionally overexploited^39^ in 1987. Pumping has occurred in ~1000 wells at rates of 24 (in 1973), 69 (in 1987), and 86 hm^3^/yr (in 2006)^38,39^, which led to a spatially variable continuous piezometric level decrease (at rates within the 0.5–10 m/yr range). Available piezometric information consists of water-level time series for a few points, from the 1970s to present. A long drought period from 1990 to 1995 (also in 1999–2000 and 2005–2007) reduced natural recharge and increased pumping in the Guadalentín Basin, which led to an increased resources deficit. All this information indicates a long-term trend in the consumption of groundwater resources.

Interferometric synthetic aperture radar (InSAR) studies, while detecting the high subsidence rates affecting the Alto Guadalentín Basin, also identified a delayed transient of nonlinear compaction of the Alto Guadalentín aquifer due to the 1990–1995 drought period^4^. This suggested a relationship between local crustal unloading and stress change on active faults bordering the basin^31^. Later work^5^ extended those studies using advanced differential InSAR (A-DInSAR) techniques to process ALOS PALSAR (2007–2010) and COSMO-SkyMed (2011–2012) radar images. The combination of multi-sensor SAR images with different resolutions allowed for a longer monitoring time span of 20 years (1992–2012) over the Alto Guadalentín Basin. Additionally, the satellite measurements provided locally comparable results with measurements acquired by two continuous GNSS stations located in the study area. Furthermore, the work presented a new soft soil thickness map and collected historical piezometric data, in order to assess aquifer system compressibility and groundwater level changes in the past 50 years. From the analysis of these data with A-DInSAR displacement measurements, the authors concluded that the governing mechanism of the Alto Guadalentín aquifer system is an inelastic, unrecoverable and delayed compaction process between water level depletion and ground surface displacement, related to the presence of very thick (>100 m) unconsolidated sediments (clay and silts).

Despite the aforementioned achievements, the previous studies focusing on the deformation in the area are based on InSAR analysis using ascending and/or descending acquisitions, without any combination of the datasets to estimate both vertical and horizontal (E-W) components^4,5^. Therefore, only the line-of-sight (LOS) displacement field is known in the Alto Guadalentín area at a regional level and it was assumed to correspond completely to vertical displacement. Although this is a common procedure in subsidence studies using InSAR measurements^40–46^, the main consequences are i) the neglecting of possible horizontal displacement components and ii) the likely overestimation of vertical displacement.

Here, while we afforded the problem on the decomposition of LOS measurements in the E-W and vertical components over the investigated area, we can provide additional constraints on the spatial and temporal evolution of the subsidence process as well as on the main governing mechanisms (e.g. temporal changes of pore pressure, geometry of the reservoir). With this primary aim, we established a GNSS network consisting of 33 stations in 2015, which densely covers the Alto Guadalentín basin (Fig. 2). This network has been observed in survey, or campaign, mode. Here we analyzed the measurements carried out in November 2015, June-July 2016 and February 2017. GNSS raw data have been processed by adopting standard processing strategies for this type of network and referred to a local reference frame in order to estimate the 3D deformation field (see Methods Section and Supplementary Information). Despite the limited time interval covered by the surveys, we estimated, for the first time, a significant 3D deformation field which is primarily related to the local exploitation of the aquifer. SAR data from the Sentinel-1 Copernicus constellation, acquired in ascending and descending orbits for the same time period, also were processed to obtain the respective LOS displacements. Using the GNSS and SAR-based deformation fields, we estimated both the vertical and horizontal components of the displacement over the entire area. In the following sections, the main results are described, compared and interpreted using the forward model and inversion technique previously mentioned and described in the Methods section.

## Results

### Global Navigation Satellite System (GNSS) results

**Figure 3:**
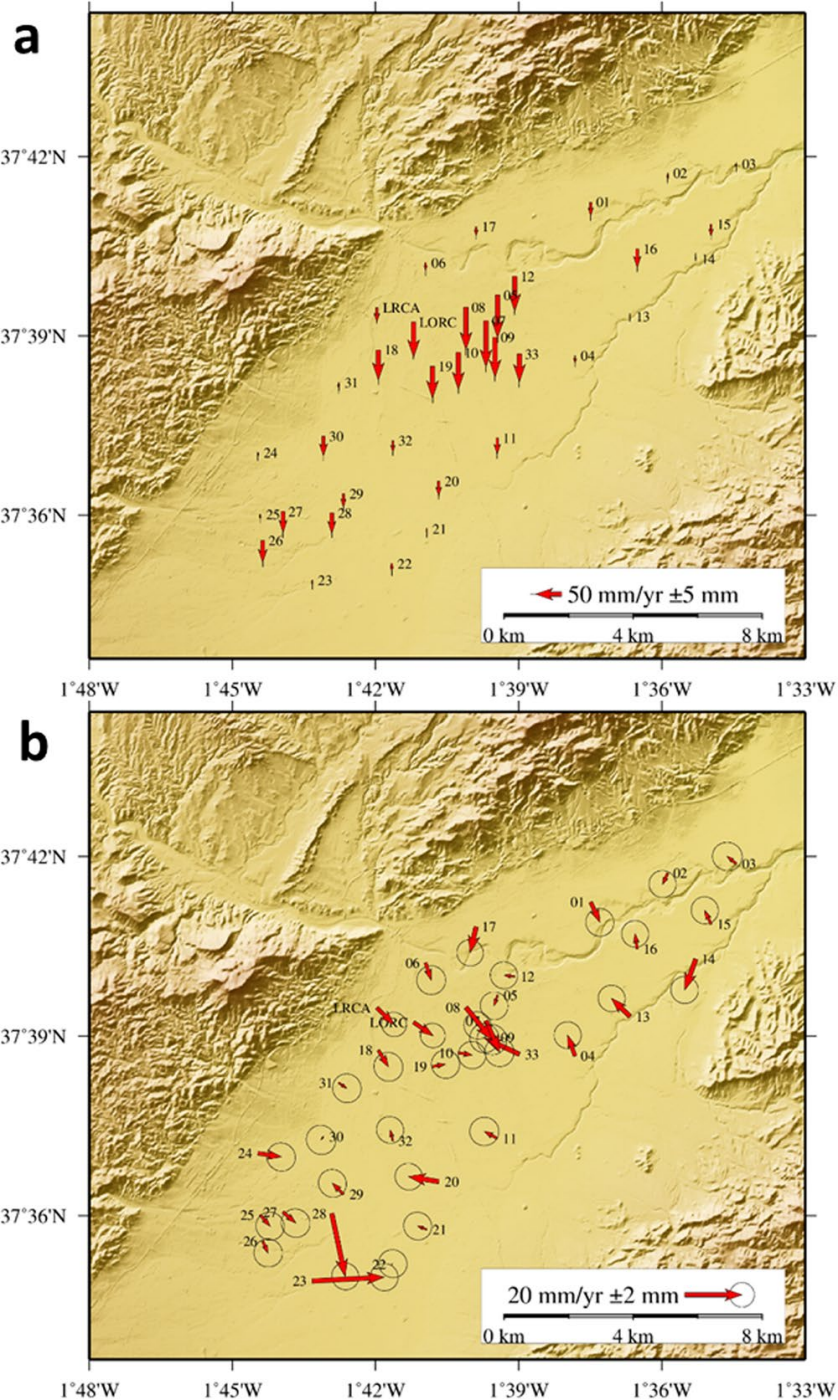
Displacement rates determined from GNSS observations. Results corresponding to the period November 2015–February 2017. (**a**) Annual vertical displacement rates, subsidence, measured with standard confidence bars. (**b**) Average annual horizontal displacements with standard confidence regions. Additional results are shown in the Supplementary Information. This figure was generated using GMT software^75^.

Three geodetic campaigns have been carried out in November 2015, June-July 2016 and February 2017. These surveys were conducted using 10 dual frequency Topcon GPS + GLONASS receivers and choke ring antennas on a four hour session basis (see Supplementary Information for details about the monuments and antennae setting characteristics). All stations were measured at least twice during the 2015 campaign and at least three times in the 2016 and 2017 campaigns with a 1 Hz sampling interval data recording (see Methods section for the description of the GNSS data processing). The 3D velocity field results are shown in Fig. 3 for both the vertical and horizontal components determined by comparing the coordinates obtained for the time spans of the three surveys. Time series for selected stations are shown in Supplementary Fig. S2.

The maximum vertical subsidence rate (9.0 ± 0.5 cm/yr) is of the same order of magnitude as that previously detected by earlier InSAR studies^4,5^. The maximum horizontal displacement rate detected is 2.5 ± 0.3 cm/yr (about 28% of the vertical displacement rate), a non-negligible amplitude. In the area showing the highest subsidence rate, again previously detected by InSAR techniques, a characteristic pattern of horizontal deformation appears (Fig. 3). These deformations, as theoretically expected, show a centripetal pattern towards the zone of maximum subsidence, located in the central part of the monitored area.

Also in the southern area, where there is a relative maximum in the LOS displacement detected by InSAR, significant horizontal motions are detected (see Fig. 3b) associated with GNSS stations 23 and 28. After comparison with A-DInSAR results and field inspection, we conclude that these are produced by very local movements related to monument instabilities (see Supplementary Fig. S3).

### Advanced Differential Satellite Synthetic Aperture Radar (A-DInSAR) results

The low revisit time (12 days per satellite, 6 days as a constellation) of the Sentinel-1 satellites, the total coverage of the European Plate, and the free availability of these products, make them an optimal choice for this study. We used the Interferometric Wide Swath (IW) mode to perform A-DInSAR processing of the Lorca area Sentinel-1A images (see Methods section for a description of the advanced processing of the satellite radar images).

Both orbits, ascending and descending (tracks 103 and 8 respectively) from Sentinel-1A, were used to decompose the measured LOS movement/mean velocities into horizontal (E-W) and vertical components^47–49^ over the studied area.

**Figure 4:**
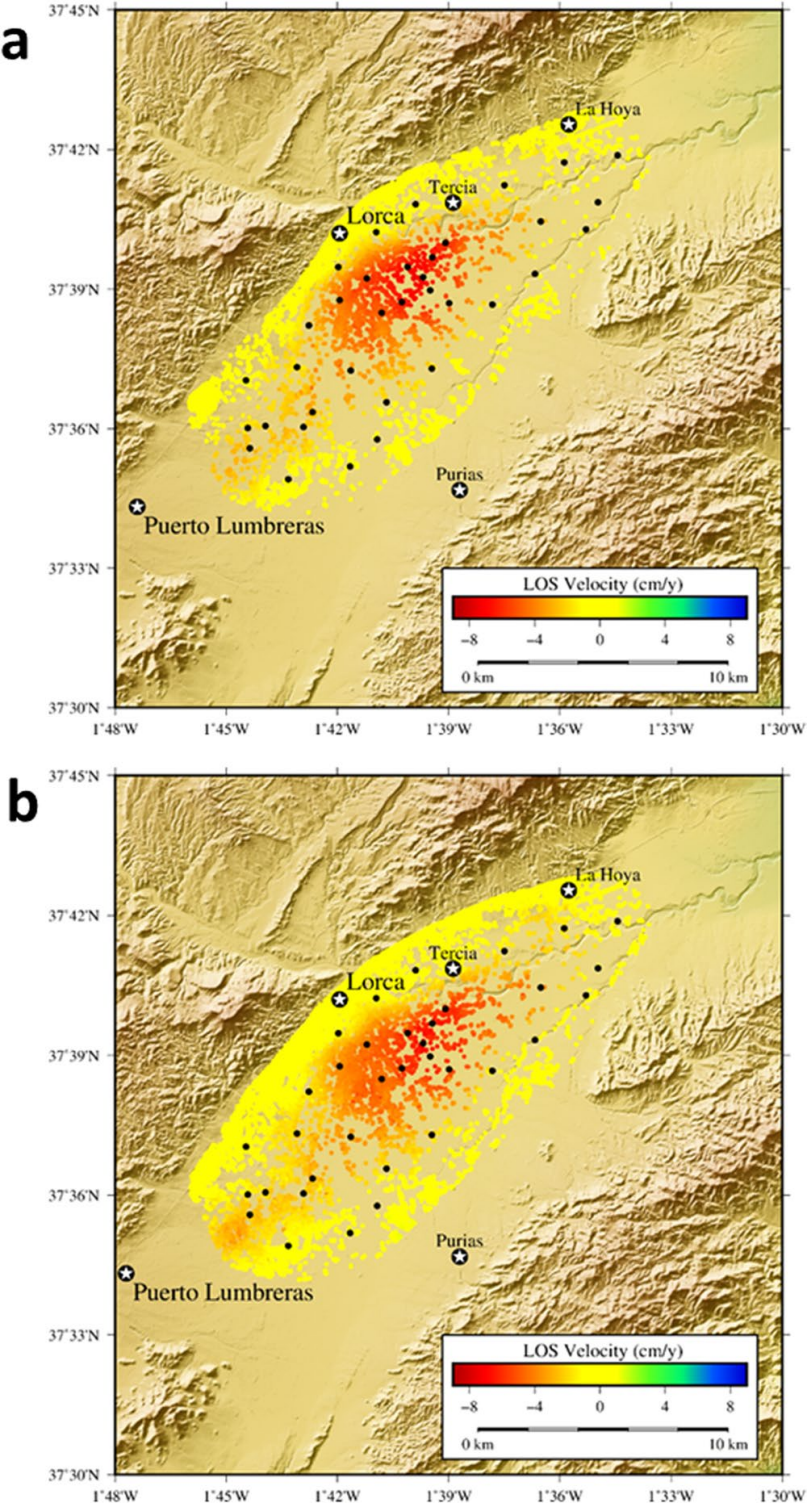
Results obtained from the A-DInSAR processing using CPT technique. Both geometries, ascending and descending, have been processed using a multilook window of 3 × 13 pixels (azimuth × range) which generates a square pixel of about 60 × 60 meters in ground resolution. Coherence method has been used for pixel selection coherence method. Results are shown for the period November 2015–February 2017. (**a**) Line of Sight (LOS) velocity values obtained for the ascending orbit. (**b**) LOS velocity values for the descending orbit. Black dots locate the GNSS stations. GMT software was used to create this figure^75^.

Our A-DInSAR study covers the same time interval spanned by the GNSS campaigns (November 2015 – February 2017). Radar data were processed using the Coherent Pixel Technique (CPT)^50^ (see Methods Section). The total area covered by the GNSS network is approximately 70 km^2^. For the A-DInSAR we processed an extended region with a total area of 170 km^2^. In both geometries, ascending and descending, the study area is covered by three bursts of the same swath. We have used a total of 42 Interferometric Wide Swath (IW) SLC images from the Sentinel-1A satellite, which results in 185 interferograms (22 images and 137 interferograms for ascending data, 20 and 48 respectively for descending; see Supplementary Tables S2 to S4). The results are shown in Fig. 4, while some selected time series are shown in the Supplementary Fig. S5 for descending LOS. This is the first InSAR study of the Alto Guadalentin Basin using two different geometries for the same time period.

Because SAR is sensitive in the perpendicular direction to its azimuth and describes an almost polar orbit, it is assumed that the detected displacement is caused by vertical and E-W motion, and the N-S motion is neglected. To obtain the vertical and E-W components of the displacement from the ascending and descending LOS motions, the following equation system must be solved^10,51^.

1 $$[\begin{array}{c}{u}_{z}\\ {u}_{ew}\end{array}]=[\begin{array}{cc}-\cos ({\theta }_{asc}) & \sin ({\theta }_{asc})\,\cos ({\alpha }_{asc})\\ -\cos ({\theta }_{dsc}) & \sin ({\theta }_{dsc})\,\cos ({\alpha }_{dsc})\end{array}]\cdot [\begin{array}{c}{u}_{los}^{asc}\\ {u}_{los}^{dsc}\end{array}],$$

where *u*_*los*_ is the displacement detected for each geometry (considered positive when it is away from the satellite and negative when it is towards the satellite), α_*asc*_ and α_*dsc*_ are the heading angles of the satellite and *θ*_*asc*_ and* θ*_*dsc*_ are the incidence angle of the SAR beam which are determined for each pixel. An additional minor correction due to the squint angle of the SAR beam can be made^51^. However, in order to apply this last correction, the coordinates of each pixel over the original SAR image are necessary and SUBSIDENCE-GUI (the software implementation of CPT) currently is unable to produce this information, so we were unable to apply this correction to our results.

**Figure 5:**
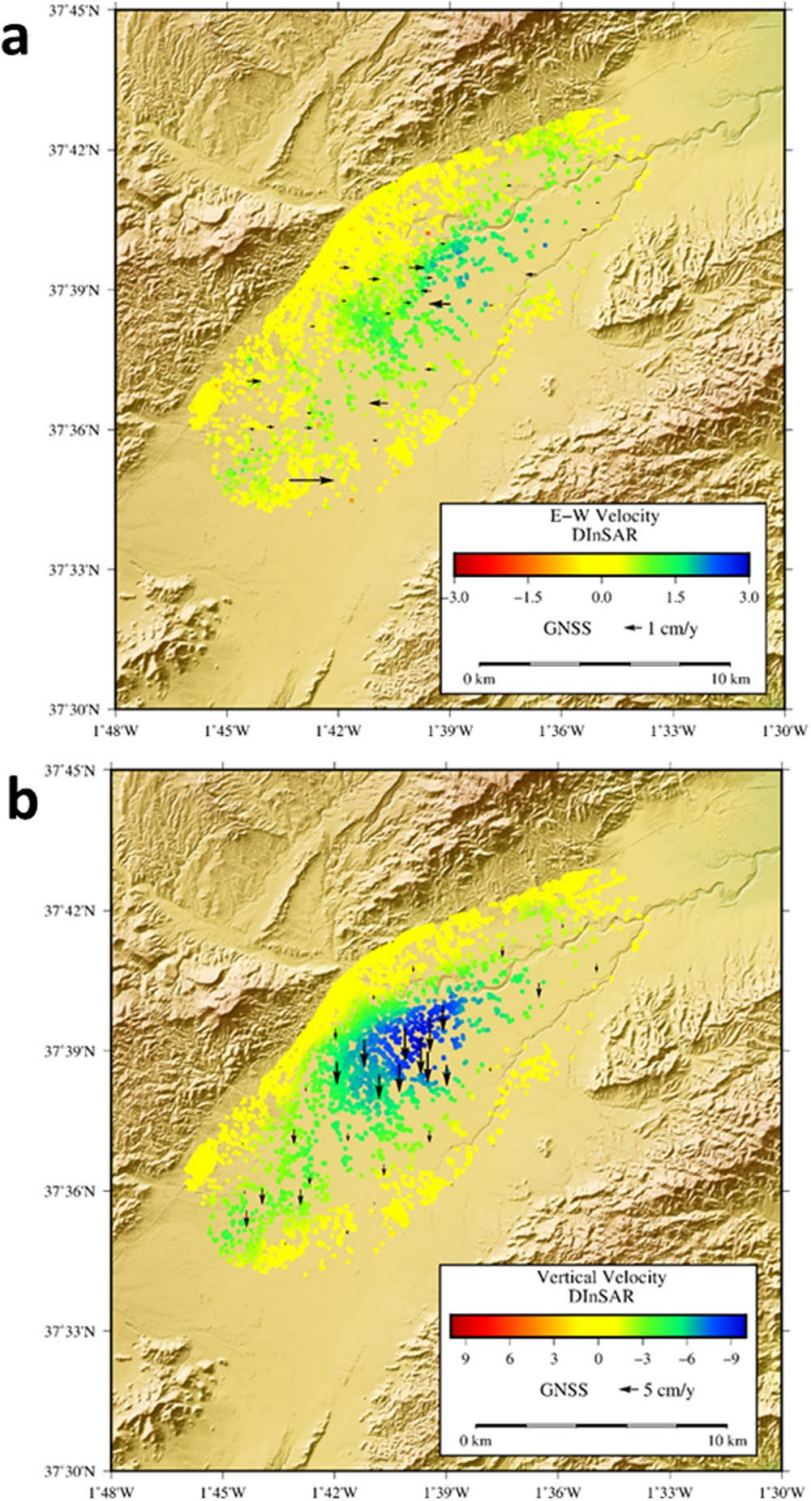
East-West and Vertical displacements obtained by A-DInSAR. (**a**) Horizontal (East-West) and (**b**) vertical (Up-Down) displacement rates estimations obtained by decomposition of the LOS detected velocity using ascending and descending orbits. GNSS displacements are also plotted with arrows to compare. Results are shown for the period November 2015 - February 2017. GMT software was used to create this figure^75^.

The decomposition into vertical and E-W displacements also introduces the need for interpolation^52–54^ because the pixels identified for ascending and descending satellite orbits are not identical in most cases. Such a decomposition is allowable only when the deformation signal is sufficiently smooth and well-sampled. Interpolation can be avoided by using the LOS data directly in the parameter estimation procedure^10,55,56^. E-W and vertical components of the displacement fields in the area obtained using ascending and descending LOS results are shown in Fig. 5.

For this case, the decomposition above does not produce particularly good results due to the previously mentioned methodological aspects and to the fact that the magnitude of the E-W motion is at sub-centimeter levels in many of the coherent pixels, i.e., the same order of the A-DInSAR uncertainty. In Supplementary Table S5 we compare GNSS and A-DInSAR results for those stations which have coherent pixels from the ascending and descending time series within 100 meters. The comparison allows us to estimate the error of the A-DInSAR processing, relative to GNSS, as ~0.7 cm in the vertical velocity component (good agreement comparable to the GNSS precision in this component) and ~1.0 cm for the E-W velocity component (worse agreement but consistent with the previously described limitations). If we project the three components of the measured displacement rates from the GNSS into the LOS (see Supplementary Table S6) and compare with the measured LOS (A-DInSAR), we obtain better results: ~0.7 cm for both ascending and descending orbits.

Other methodologies that can be used to obtain the North-South component of the displacement, such as Pixel Offset Tracking or Multiple Aperture Interferometry, were considered but discarded since the magnitude of the displacement in this component is not enough to obtain a reliable result with those or other techniques^57–61^. Multi-platform and multi-angle InSAR-driven combination methods, such as Multidimensional Small Baseline Subset (MSBAS)^62^, could increase the temporal span in the InSAR time-series but there are no GNSS data available for comparison during those time periods.

## Discussion

As previously noted, the establishment and observation (spanning November 2015 - February 2017) of a local GNSS network allows, for the first time, for measurement of the 3D displacement field in the Alto Guadaletin area, associated with exploitation of the local aquifer. Also, for the first time, A-DInSAR results have been obtained using both ascending and descending radar images from the Sentinel-1A Copernicus radar satellite, allowing estimation of both vertical and horizontal (E-W) displacement components, a 2D displacement field, at higher spatial resolution than GNSS. See Figs 3 to 5.

Our results highlight how the ad hoc establishment of survey mode GNSS networks improves the spatio-temporal monitoring of the 3D displacement field of areas subjected to extensive groundwater extraction, therefore representing a valuable monitoring technique. Moreover, GNSS observation provides complementary information to A-DInSAR results, allowing for their validation and scaling. In addition, at a local level, it is observed that the GNSS network does not completely cover the current displacement area, in particular along the SW region (see Figs 4 and 5), because the network was defined based on displacements obtained prior to 2012 (see Fig. 1). But our Sentinel-1 A-DInSAR results show that the deformation has extended in the SW direction, which today is the region of the most significant water extraction^63^. Therefore, the GNSS network needs to be extended over that region with additional GNSS stations.

Our results for the studied area highlight that: (i) simultaneous GNSS and A-DInSAR results are consistent with each other (Fig. 5 and Supplementary Tables S5 and S6); (ii) the results obtained for rates and pattern of the displacement are consistent with previous DInSAR results^4,5^; however, (iii) the horizontal displacement rate has a maximum amplitude of 2–3 cm/year (Figs 3 and 4) and it is a significant component of the observed deformation field. Therefore, the horizontal displacement cannot be neglected, as in the discussion and interpretation sections of previous studies^4,5^.

Because the results here demonstrate that the horizontal displacements represent a significant component of the deformation field of the studied area, we also performed sensitivity tests by neglecting/including this horizontal motion in order to assess the variability (or bias percentage) on the determination of the aquifer characteristics and their temporal evolution using deformation modeling. To do this, we employed our GNSS and A-DInSAR results. Also, taking into account the linear time behavior of the displacement field (see Supplementary Figs S2 and S5), we considered displacement rates in our study.

We employed four different data sets (Cases) of surface displacement covering the period November 2015 to February 2017, and we carried out the inversion using the described forward model and inversion methodology (see introduction and Methods section). In the Supplementary Information (pages 13–17) we describe a complementary study carried out considering ten Cases, which have been obtained by combining the available and different data sets. Here it is clearer to show only the most representative ones, to demonstrate the main consequences of neglecting horizontal displacements on the resulting interpretation. Subsequently we evaluate the consequences and implications for operative monitoring at a global scale (see the Supplementary Information study for additional details).

The cases described here are the following:

(A) LOS A-DInSAR results obtained for descending orbit images, assuming 100% as vertical displacement.
(B) Purely LOS A-DInSAR results obtained for descending orbit images.
(C) Purely LOS A-DInSAR results obtained for ascending and descending orbit images.
(D) Purely LOS A-DInSAR results obtained for ascending and descending orbit images together with the 3D displacements determined using the GNSS surveys results.

Case **A** is one-dimensional (1D), **B** and **C** are 2D (indirectly by combining Up-Down and E-W in the measured LOS), and **D** is a combination of 2D and 3D data (2D + 3D data).

**Figure 6:**
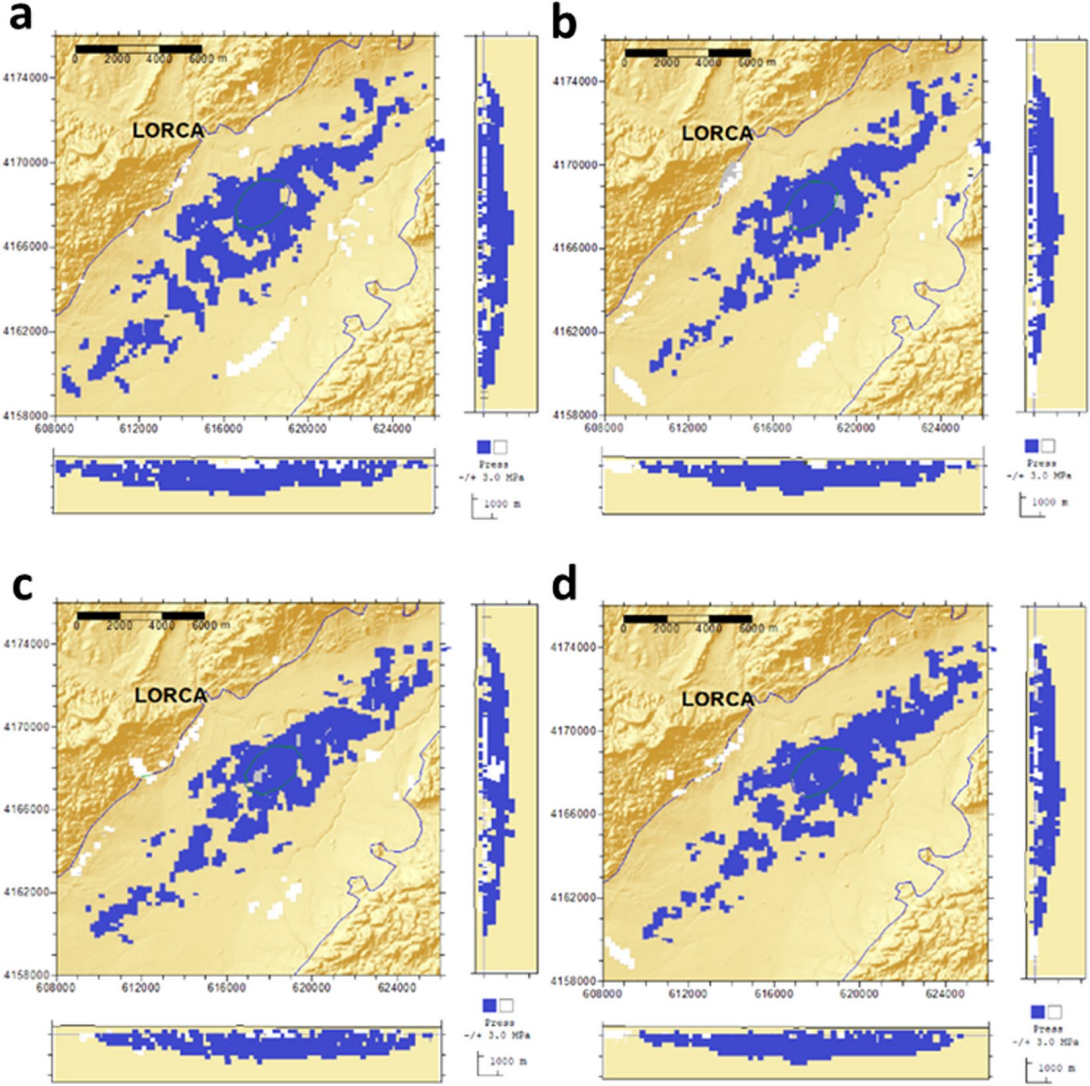
Representation of the inversion results obtained for the 1D, 2D and 2D + 3D considered data sets. (**a**) Obtained source for Case **A**; (**b**) for Case **B**; (**c**) for Case **C**; and (**d**) for Case **D**. Blue color indicates negative pressure value cells, produced by water extraction. White color indicates positive pressure change cells. These positive pressure sources adjust the errors and the effects of other deformation sources, different from water extraction (e.g., of tectonic origin). This figure was created using Surfer 8.02 Surface Mapping System (www.goldensoftware.com/products/surfer) and Paint, Microsoft Windows 10.

We invert each case and estimate the volume changes of the water table (volume and geometry) assuming a given pressure change value. Moreover, based on hydrogeological observations, we impose the criteria that sources are shallower than one kilometer. A summary of the results is provided in Table 1 and Fig. 6. In Fig. 6, the blue colors indicate negative pressure values, while white colors indicate positive pressure change cells. The former are related to the loss of pore pressure due to aquifer overdrawing, the latter are related to modelling of measurement errors and/or effects related to other deformation sources (e.g., of tectonic origin). Noting this, it is interesting to observe how the cells with positive pressure changes tend to accumulate along directions of faults existing in the area^36^ (see Figs 1 and 6). This potentially indicates some relation with the thickness of the compacting material across the fault. While this is outside the scope of this study, these results suggest that additional research in these aspects should be carried out in the future, and that next models of the observed deformation should introduce additional sources.

**Table 1:** Numerical summary of the inversion results obtained for selected cases.

CASE	Intensity (MPa × Km^3^)	Misfit (cm)	Mean Model Intensity (MPa × Km^3^)	Pres. (MPa)	Vol. (Km^3^)	Displacement components considered	Number of data used
A	−41	0.36	−41	−3	13.7	1D	1505
B	−32	0.30	−33	−3	11	2D	1505
C	−33	0.32					2708
D	−34	0.43				2D + 3D	2816

Inversion results include the volume and geometry of the active part of the aquifer which has produced the measured displacements. Here this is quantified by the intensity, which is equal to the product of volume by pressure change; it is impossible to determine both quantities separately. If we increase pressure, we decrease volume and vice versa. Here, in order to determine a general geometry, we have constrained the value of the pressure change^30–32^. We consider a pressure value of −3 MPa, after a trial analysis, selecting the value that gives us a source geometry most consistent with the characteristics of the aquifer.

Note that the inversion results obtained from these data sets can be organized into two subsets: (i) Case **A** (1D, vertical displacement) and (ii) Cases **B**–**D** (2D and 2D + 3D). Results for group (ii) are internally very consistent, with scattering on the order of 3% (Table 1).

The results of (i), 1D results, are ~24% greater in intensity/volume than those of (ii) (2D and 2D + 3D results), indicating that using only one component of the displacement field and assuming that displacements are only vertical significantly overestimates the volume of water extracted during the study period (on the order of tens of hm^3^). This can have an important effect in predictions of future volume variations and surface displacements.

Another important result is that we do not observe significant differences (3–4%, at the level of error or lower, see Table 1 and Supplementary Information) between using just one LOS (ascending or descending), both LOS (ascending and descending) displacement data sets together, or both combined with the GNSS results. The estimation of source characteristics is very similar for all cases, just slightly changing the misfit of the minimum values for Cases **B–D**.

In summary, contrary to previous studies in the Lorca area, the measurement and use of the horizontal and vertical displacements at the surface is important for the prediction of future volume variations and surface displacements. These differences can have important effects on the design of monitoring systems, help in the decision-making process related to the sustainable management of the aquifer resources, and improve the assessment of potential hazards related to the aquifer exploitation.

Our main conclusions, summarized above, do not include any local assumptions which could condition our interpretation methodology, but they have general applicability worldwide. The Lorca case can be considered an extreme case, taking into account that it has significant E-W horizontal deformation, but only in geographically limited areas of maximum deformation. In other regions, where significant horizontal E-W deformation may be more scattered and cover more extended areas (see the synthetic test case in the Supplementary Information, text, Figs S8 and S9, and Table S8) the effect of considering vertical deformation alone could be even more dramatic.

We have shown, using inversion results from different data sets, that the operational monitoring of the aquifer can be done using A-DInSAR with ascending and/or descending satellite radar images. Considering the inversion results described previously and in the Supplementary Information, the most effective method is to carry out a joint inversion of LOS measurements determined using ascending and descending radar images. GNSS, using continuous or survey observation mode, can be used for validation and scaling purposes. GNSS stations should be installed in those locations that will best constrain the A-DInSAR results in areas of zero deformation for reference, maximum deformation areas for scaling and study of the time variation, or in low coherence areas so that both techniques complement each other. Continuous GNSS observations are preferable, if possible. The proposed methodology will potentially reduce the cost of the geodetic monitoring system in a very important way.

In addition, this effective A-DInSAR monitoring can be accomplished with the freely-available Copernicus Sentinel-1A and -1B satellite data, considering their global coverage and repeatability, ensuring their effective use for monitoring on a global scale.

## Methods

### Global Navigation Satellite System (GNSS)

Raw GNSS observations collected on the episodic geodetic network were processed using GAMIT/GLOBK 10.6 software^64^. To improve the overall configuration of the network and tie the local measurements to a regional reference frame, data coming from more than 20 continuous stations belonging to regional (REGAM and MERISTEMUM), wide-scale (IGNE and EPN) networks were introduced in the processing (see Supplementary Information for additional details). In a first step, we used daily double-differenced GNSS phase observations, on a 30-sec sampling basis; the observations were weighted according to the elevation angle, for which a cut-off angle of 10° was chosen. In addition, we used the latest absolute receiver antenna models by the IGS and we adopted atmospheric zenith delay models^65^, coupled with the Global Mapping Functions for the neutral atmosphere. The results of this processing step are daily estimates of loosely constrained station coordinates, and other parameters, along with the associated variance-covariance matrices. In a successive step, the loosely constrained daily solutions were used as quasi observations in a Kalman filter (GLOBK) in order to estimate a consistent set of daily coordinates (i.e. time series) for all sites involved. Each time series was analyzed for linear velocities and antenna jumps; in order to obtain clean time series, any position estimate whose uncertainty was greater than 20 mm or whose value differed by more than 10 mm from the best-fitting linear trend was removed. In a final step, all loosely constrained daily solutions and their full covariance matrices were combined to compute a set of coordinates and velocities related to ITRF2008 geodetic reference frame^66^. In this step, to account for correlated errors, we added a random walk component^62^ of 1.5 and 2.5 mm yr^−0.5^ to the assumed error in horizontal and vertical positions, respectively. To adequately show the crustal deformation pattern over the studied area, i.e. to isolate the local deformation field from the regional tectonic pattern, we rotated our estimated GNSS velocities to a local reference system defined by the minimization of the long-term velocities^67^ of ALAC, ALBA and ALME continuous stations from EPN^68^.

### Advanced Differential Synthetics Aperture Radar Interferometry (A-DInSAR)

The dataset used in this study is composed of 37 Sentinel-1A Single Look Complex (SLC) images (19 ascending from track 103 and 18 descending from track 8) that were processed using SUBSIDENCE-GUI interferometric software. Prior to the interferometric generation, all images for each orbit were registered to a common master image. The master image was selected to minimize perpendicular and temporal baselines across the dataset in order to avoid registration errors.

Interferogram pairs were generated by a double minimum criteria, avoiding those with high temporal and perpendicular baselines. This selection mode proved to generate better velocity estimation as well as a lower sensitivity to Digital Elevation Model (DEM) errors. A total of 185 interferograms (137 for ascending orbit and 48 for descending), have been generated (see Supplementary Table S3 for the entire list). To remove the topographic phase from the interferograms, an external high-resolution DEM have been used. Since there are missing acquisitions from some orbits, our study covers a temporal span from October 2015 to February 2017 which covers the period of the GNSS surveys.

To obtain the surface displacements we use SUBSIDENCE-GUI, the software implementation of the Coherent Pixel Technique algorithm, CPT^50^. This method works with distributed scatters at low-resolution over the multi-looked interferograms, similar to the wide-used Small Baselines Subset (SBAS)^69^. Because of the characteristics of the ground surface in Lorca (mostly agricultural and bare soil), this kind of analysis is more suitable than a full-resolution approach like Point Scatters (PS) method. For the two geometries (ascending and descending ones) the principal parameters of the processing have been preserved. A mean coherence map has been processed to establish a pixel selection by means of coherence, using a multilook window of fifteen samples in range and three lines in azimuth. This multilooking results in low resolution pixels obtained from an average of 45 pixels from the original interferogram, which correspond to a square pixel in a ground resolution of about 60 m × 60 m.

To select those pixels with enough phase quality to obtain surface deformation, a coherence criterion has been chosen. A threshold of a medium coherence of 0.4 corresponding to a phase standard deviation of 18° degrees has been used^70^, this value provides good spatial coverage and enough phase quality to obtain a convergent solution. A Delaunay triangulation between pixels is used, and to reduce the atmospheric artifacts in the lineal processing a limit of 800 m among pixels is also used. To estimate the linear velocity CPT needs velocity and DEM error seeds, points with known velocity and known altitude for the entire studied period^50^. For velocity seeds, several points outside of the main deformation area have been selected and for DEM seeds, large human-made flat zones were used, such as roads or parking lots (see Supplementary Fig. S4).

For the non-linear velocity estimation, the atmospheric contribution to the phase must be calculated. To filter the atmospheric perturbations, two filters were applied: a spatial low pass filtering with a 1-km correlation window and a high pass temporal filtering with a window of 60 days and a minimum of 4 samples. After this processing, the non-linear displacement can be calculated^71^.

### Direct Modeling and Inverse Technique

Considering the linear theory of poroelasticity^72^, the horizontal and vertical components (*du*, *dv*, *dw*) of the movements at a point (*X*, *Y*, *Z*) of the free surface, due to a differential nucleus, located at (*x*, *y*, *z*), with sides *dx*, *dy*, *dz*, corresponding to the reservoir with local overpressure *Δp* are^24^:

2 $$(\begin{array}{c}du\\ dv\\ dw\end{array})={\rm{\Delta }}p\frac{1-\nu }{\pi }{c}_{m}\,(\begin{array}{c}X-x\\ Y-y\\ Z-z\end{array})\,\frac{dxdydz}{{({(X-x)}^{2}+{(Y-y)}^{2}+{(Z-z)}^{2})}^{3/2}}$$

where *ν* denotes Poisson’s ratio (≈0.25), *c*_*m*_ the uniaxial compaction coefficient. Assuming that displacements at the surface happen to be almost directly proportional to the thickness Δ*z* of the reservoir, the volume integrations for a parallelepiped cell of sides *Δx*, *Δy*, *Δz* and overpressure *Δp* in equation (2) can be simplified to integration in the horizontal plane only given rise to^24^:

3 $$(\begin{array}{c}du\\ dv\\ dw\end{array})={\rm{\Delta }}p\,\frac{1-\nu }{\pi }\,{c}_{m}I\,{\rm{\Delta }}z$$

where:

4 $$\begin{array}{rcl}I & = & {I}_{i}(X-(x+{\Delta }x/2),Y-(y+{\Delta }y/2),Z-z)\\  &  & -\,{I}_{i}(X-(x+{\Delta }x/2),Y-(y-{\Delta }y/2),Z-z)\\  &  & -\,{I}_{i}(X-(x-{\Delta }x/2),Y-(y+{\Delta }y/2),Z-z)\\  &  & +\,{I}_{i}(X-(x-{\Delta }x/2),Y-(y-{\Delta }y/2),Z-z)\end{array}$$

The integrals *I*_*i*_ for the displacements in the *i*-direction are:

5 $${I}_{z}(p,q,r)=\frac{1}{2}\frac{p}{|p|}\{arcsin\,\frac{{p}^{2}{q}^{2}-{r}^{2}({p}^{2}+{q}^{2}+{r}^{2})}{({p}^{2}+{r}^{2})\,({q}^{2}+{r}^{2})}+\frac{\pi }{2}\}$$

6 $${I}_{x}(p,q,r)=arcsinh\frac{p}{\sqrt{{q}^{2}+{r}^{2}}}$$

7 $${I}_{y}(p,q,r)=arcsinh\frac{q}{\sqrt{{p}^{2}+{r}^{2}}}$$

This formulation provides the direct calculation of the surface effect of a single parallelepiped cell. The total effect of an anomalous structure described as aggregation of *m* small parallelepiped cells is obtained, according^24^, as addition of the partial effects. This direct formulation can be used to carry out the inverse approach in order to determine the pressure 3D source structure responsible of the observed surface deformations.

Camacho^30^ presented an original methodology for simultaneous inversion of three dimensional displacement data, *LOS*, or any combination of terrestrial and space displacement data, by means of 3D extended bodies with free geometry for anomalous pressure. The approach determines a general geometrical configuration of pressurized sources corresponding to prescribed values of anomalous pressure. These sources are described as a 3D aggregate of (thousands of) pressure elemental sources, and they fit the entire data set within some regularity conditions. The approach works in a step-by-step growth process that allows us to build very general geometrical configurations.

The observation equations are:

8 $${\boldsymbol{ds}}={\boldsymbol{d}}{{\boldsymbol{s}}}^{{\boldsymbol{c}}}+{\boldsymbol{v}}$$

where ***ds***, ***ds***^***c***^ represent the vector of observed and calculated three component (3D) deformations, and ***v*** is the vector for residual values coming from inaccuracies in the observation process and also from insufficient model fit. In that methodology surface deformation, ***ds***^***c***^, due to a buried over pressure structure is computed as the aggregated effect for several point sources, as due to the deformation effects from the incremental pressure *p*_*k*_ and expansion radius within the elastic semi space, originally formulated as a Mogi model^73^. In this work we substitute this source with the poroelastic expressions^24^ for 3D reservoirs according the preceding formulation for parallelepiped cells.

The inversion equations (8) are solved by means of adding a regularization misfit conditions

9 $${{\boldsymbol{v}}}^{T}{{\boldsymbol{Q}}}_{D}^{-1}{\boldsymbol{v}}+\lambda \,{{\boldsymbol{m}}}^{T}{{\boldsymbol{Q}}}_{M}^{-1}{\boldsymbol{m}}=\,{\rm{\min }}\,.$$

where model vector ***m*** is constituted by the values of pressure × volume, *m*_*k*_ = *p*_*k*_*∆x*_*k*_*∆y*_*k*_*∆z*_*k*_, *k* = *1*, …, *m*, for the *m* cells of the model, ***Q***_***D***_ is a covariance matrix for the data, ***Q***_***M***_ is a suitable covariance matrix corresponding to the physical configuration and *λ* is a smoothing factor for selected balance between fitness and smoothness of the model. The inversion approach is a non-linear problem.

The anomalous source is determined as a free aggregation of a large number of small sources with anomalous pressure. We carry out a step-by-step process of growth of the 3D models, using an exploratory technique to find each new cell to be filled with anomalous pressure values and aggregated to the models.

## Electronic supplementary material

Supplementary Information

## Data Availability

Sentinel-1 data can be downloaded through the Copernicus Open Data Hub. The datasets generated during and analysed during the current study are available from the corresponding author on reasonable request.
