# Supplementary material for: Modeling the two- and three-dimensional displacement field in Lorca, Spain, subsidence and the global implications
Source: Sci Rep. 2018 Oct 3;8:14782. doi: 10.1038/s41598-018-33128-0 (PMC6170491; doi:10.1038/s41598-018-33128-0)
Supplement: Supplementary file 1 — Supplementary Information [file 41598_2018_33128_MOESM1_ESM.pdf]

Supplementary Information

Modeling the two- and three-dimensional displacement field in Lorca, Spain, subsidence and the global implications.

Jose Fernandez[1,*], Juan F. Prieto[2], Joaquin Escayo[1], Antonio G. Camacho[1], Francisco Luzón[3], Kristy F. Tiampo[4], Mimmo Palano[5], Tamara Abajo[1], Enrique Pérez[6], Jesus Velasco[2], Tomas Herrero[6], Guadalupe Bru[1], Iñigo Molina[2], Juan López[6], Gema Rodríguez-Velasco[7], Israel Gómez[1], Jordi J. Mallorquí[8]

[1]Instituto de Geociencias (CSIC, UCM). Calle del Doctor Severo Ochoa, nº 7. Facultad de Medicina (Edificio Entrepabellones 7 y 8, 4ª planta). Ciudad Universitaria. 28040-Madrid, Spain.

[2]ETSI Topografía, Geodesia y Cartografía. Universidad Politécnica de Madrid. Ctra. Valencia km 7 - 28031 Madrid, Spain.

[3]Departamento de Química y Física. Universidad de Almería. Edificio CITE-IIA. Cañada de San Urbano s/n. 04120, Almería. Spain.

[4]Cooperative Institute for Research in Environmental Sciences (CIRES), 216UCB, University of Colorado at Boulder, Boulder, CO, 80309, USA.

[5]Istituto Nazionale di Geofisica e Vulcanologia, Osservatorio Etneo, 95125, Catania, Italy.

[6]Dpto. Ingeniería Agroforestal. ETSI Agronómica, Alimentaria y de Biosistemas. Universidad Politécnica de Madrid. Avda. Puerta de Hierro, nº 2 – 4. 28040-Madrid, Spain.

[7] Dpto. Física de la Tierra y Astrofísica, Unidad Departamental Astronomía y Geodesia. Universidad Complutense de Madrid, Fac. C. Matemáticas, Plaza de Ciencias, 3. 28040-Madrid, Spain

[8]CommSensLab, Dep. Signal Theory and Communications, Universitat Politècnica de Catalunya (UPC), D3-Campus Nord-UPC, C. Jordi Girona 1-3, 08034 Barcelona, Spain

---

*Correspondence and request for materials should be addresses to J.F. (email: jft@mat.ucm.es, phone: +34-913944632)

Global Navigation Satellite System (GNSS) network

In order to perform spatio-temporal monitoring of the 3D displacement field in the Lorca subsiding area, a GNSS network was designed and implemented in 2015. This geodetic network consists of 33 survey-mode GNSS stations covering the Alto Guadalentin Basin area of about 70 km^2 (see Figure 2), the region also studied by InSAR (Gonzalez and Fernández, 2011; González et al., 2012; Bonì et al., 2015).

Special care has been taken regarding the stability of the monitoring sites during the design phase of the network. Monuments of 31 of these stations consist on reinforced concrete cabinets originally settled for irrigation control sensors (see Figure S1). Each cabinet, having dimensions of 2 x 0.6 x 2 meters (for length, width and height, respectively), rests on a reinforced concrete slab (min. 40 cm thick) anchored to the floor. These cabinets have been equipped with a forced centering system for the GNSS antenna to guarantee repeatability in the reoccupation during different campaigns with accuracy usually below 0.5 mm (Figure S1a). The remaining two stations have been located on existing geodetic pillars previously built in the area. These two pillars also have GNSS antenna centering devices (Figure S1b).

Raw GNSS observations collected on this geodetic network were processed using GAMIT/GLOBK 10.6 software and taking into account precise ephemerides from the IGS (International GNSS Service; http://igscb.jpl.nasa.gov) and Earth orientation parameters from the International Earth Rotation Service (www.iers.org). As mentioned in the main text, to improve the overall configuration of the network and tie the local measurements to a regional reference frame, data coming from more than 20 continuous stations belonging to regional (REGAM and MERISTEMUM) and wide-scale (IGNE and EPN) networks were introduced in the processing. See Table S1 for details on used continuous stations.

a b

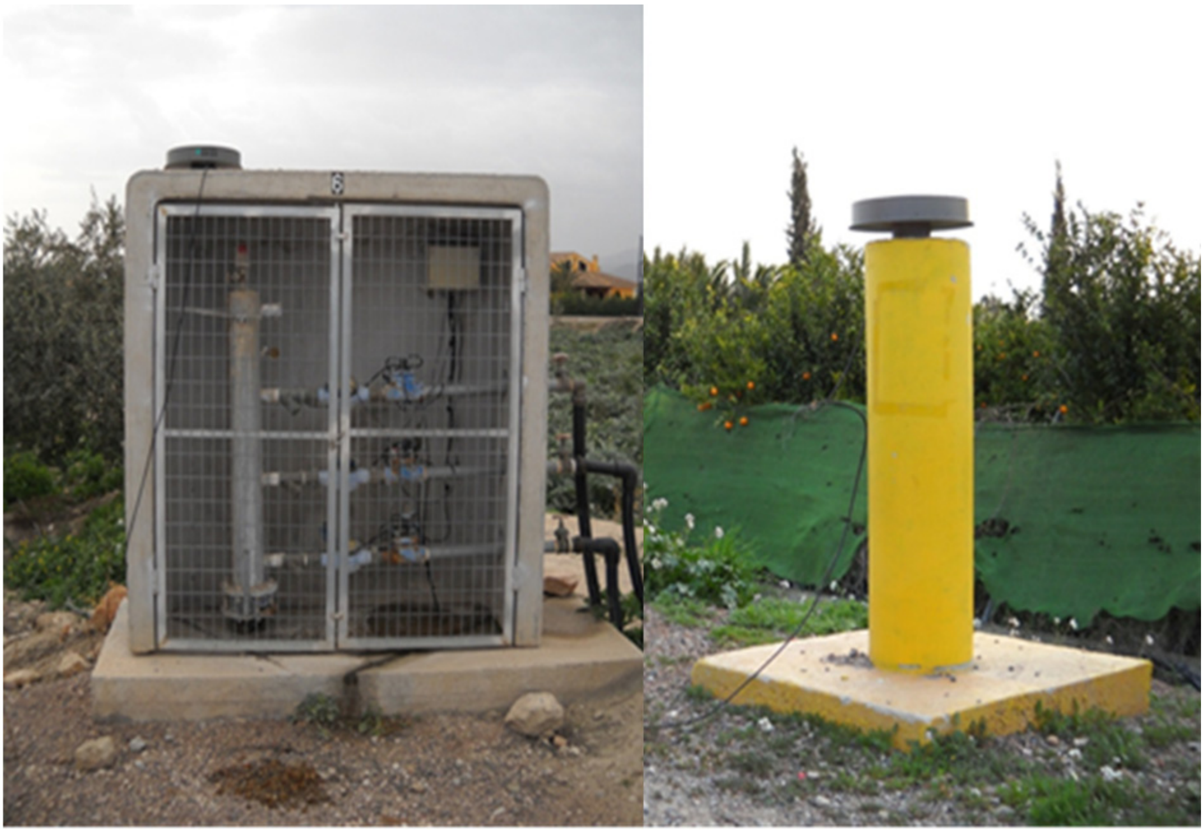

Figure S1 | Monument types adopted for GNSS stations. (a) Concrete cabinet anchored to reinforced concrete slab and **(b)** concrete pillar. The GNSS antenna is mounted on a fixed centering device to the top of the cabinet and pillar. Photographs by authors.

Site Id	Long	Lat	Ve	Vn	Ve(1σ)	Vn(1σ)	RHO	Up	Up 1σ	NETWORK
ABAN	358.94633	38.17506	20.01	17.66	0.18	0.16	0.004	1.57	0.46	REGAM
ALCA	359.1392	37.73077	20.42	17.23	0.11	0.11	0.003	0.65	0.40	REGAM
AIRM	358.87139	37.80653	20.00	15.50	0.35	0.38	0.020	0.13	1.49	REGAM
ALAC	359.51877	38.33892	19.81	16.61	0.07	0.06	0.003	0.63	0.21	EPN
ALBA	358.1436	38.97792	18.45	16.67	0.14	0.13	0.000	-9.26	0.97	EPN
ALME	357.54056	36.85254	18.52	16.15	0.07	0.07	0.001	0.84	0.39	IGNE
CABO	359.3016	37.63087	20.74	17.12	0.14	0.20	0.005	2.55	0.95	MERISTEMUM
CARG	359.02615	37.5966	---	---	---	---	---	---	---	IGNE
CARA	358.03232	38.04588	19.30	16.36	0.14	0.14	0.004	0.57	0.52	REGAM
CIEZ	358.61914	38.23316	18.63	17.03	0.29	0.26	0.009	-0.15	0.85	REGAM
CRTG	359.02072	37.60664	19.20	17.27	0.18	0.16	0.005	-1.22	0.55	MERISTEMUM
CRVC	358.13136	38.11459	18.64	16.97	0.11	0.30	0.001	0.28	0.42	MERISTEMUM
JUMA	358.67436	38.50207	19.96	16.81	0.14	0.12	0.002	0.27	0.44	REGAM
JUMI	358.67285	38.47122	19.49	15.96	0.11	0.17	0.002	-1.46	0.37	MERISTEMUM
LORC	358.31323	37.65389	27.89	8.82	0.27	0.48	0.001	-86.17	1.60	MERISTEMUM
LRCA	358.30043	37.65791	---	---	---	---	---	---	---	REGAM
MAZA	358.68951	37.59344	19.93	18.36	0.17	0.12	0.004	1.64	0.42	REGAM
MCIA	358.87748	37.99016	20.26	17.16	0.11	0.14	0.002	1.57	0.40	REGAM
MRAT	358.00118	38.24753	19.97	17.04	0.19	0.26	0.002	-0.27	0.57	REGAM
MUL1	358.55116	38.04111	19.54	16.71	0.13	0.12	0.003	0.45	0.42	REGAM
MURC	358.87532	37.99217	19.77	16.73	0.10	0.11	0.001	-0.97	0.40	MERISTEMUM
SALI	359.22147	37.83491	20.71	16.89	0.13	0.15	0.003	-0.47	0.64	MERISTEMUM

Table S1. Site coordinates and long-term velocities (referred to the ITRF2008 reference frame) of the continuous stations used in the processing. Ve and Vn are the east and north components of the site velocities in mm/yr. Uncertainties are within the 1-σ confidence level. No long-term velocity estimations, referred to ITRF2008, are currently available for LRCA and CARG stations. To adequately show the crustal deformation pattern over the studied area, as explained in the main text, we rotated our estimated GNSS velocities to a local reference system defined by the minimization of the long-term velocities of ALAC, ALBA and ALME continuous stations, externally located with respect the study area.

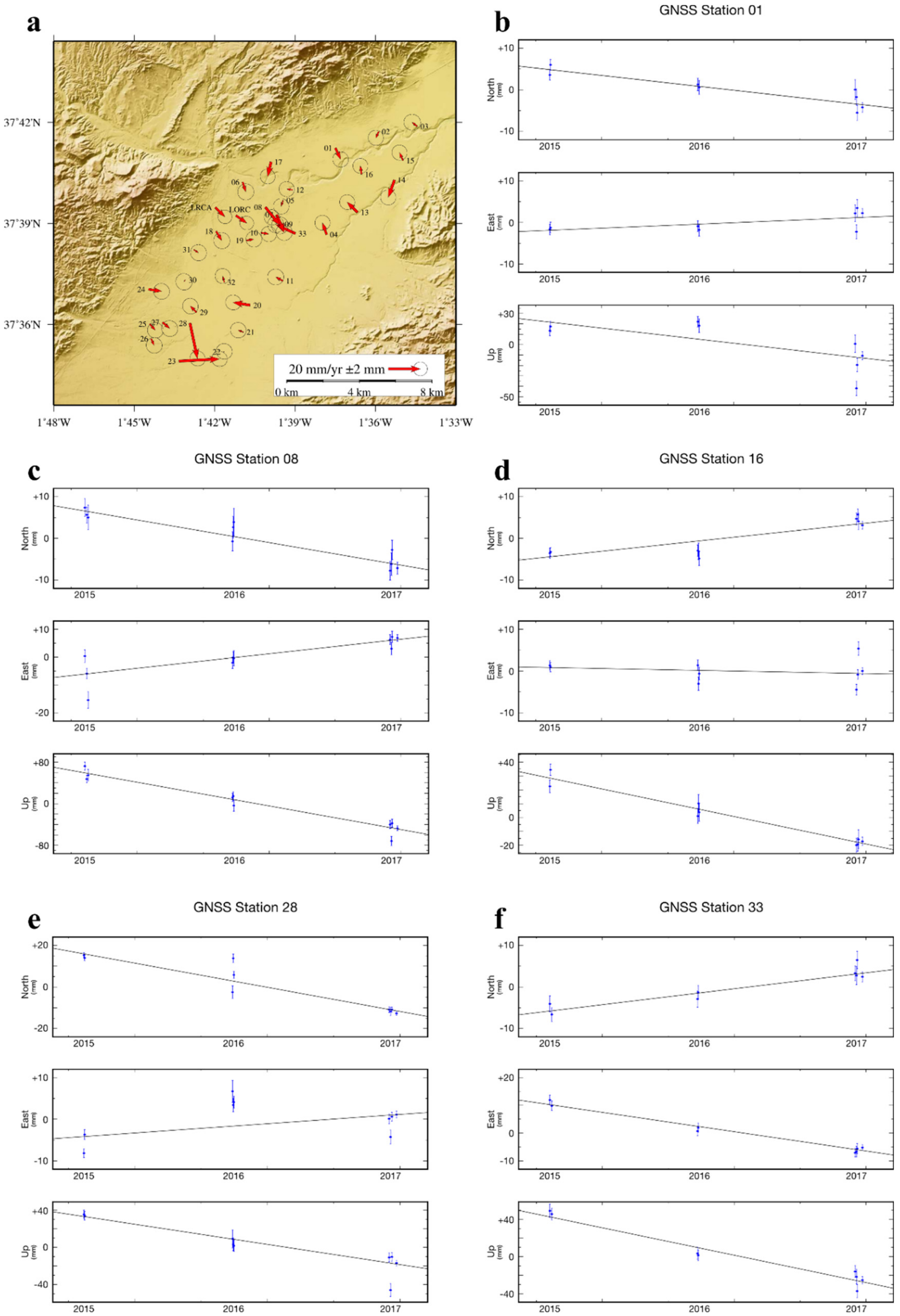

Figure S2 | Time series determined from GNSS observations for selected stations. Results correspond to the period November 2015-February 2017. **(a)** Average annual horizontal displacements with standard confidence regions. **(b)-(f)** 3D time series displacements for some selected stations representing the variety of cases. Most stations show a clearly linear behavior. Only some components in limited stations do not show linear trends. GNSS station 28 has an anomalous horizontal displacement [see panels (**a**) and (**e**), Figure S4 and main text]. GMT software (Wessel et al., 2013) has been used to generate panel **(a)** of this figure; the remainder of the panels were generated using Inkscape software (https://inkscape.org/en/).

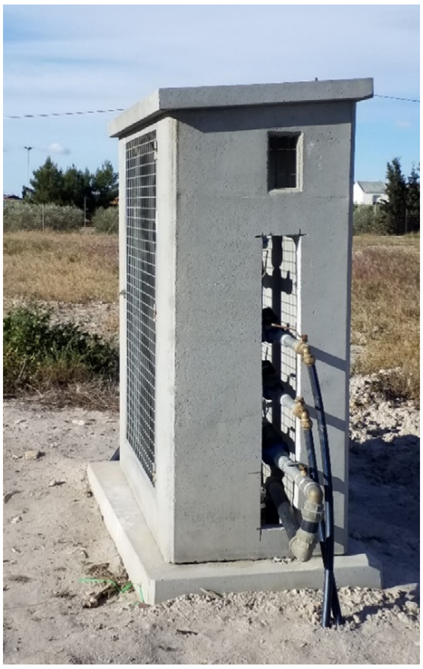

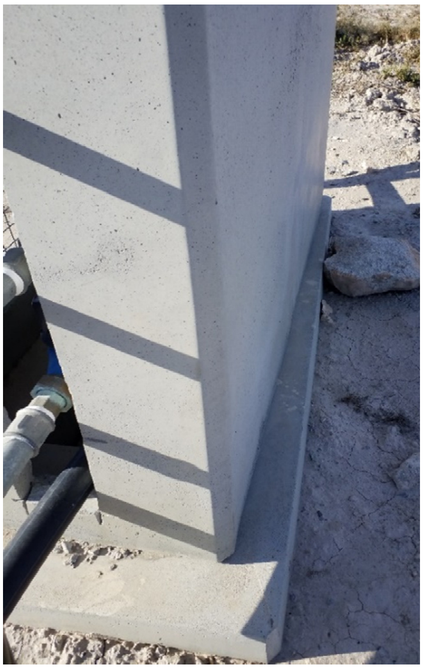

(a) (b)

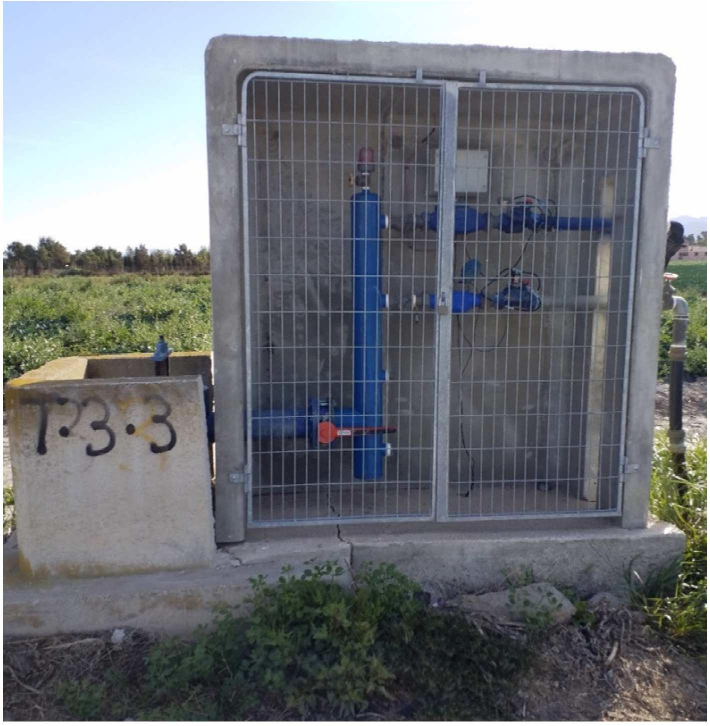

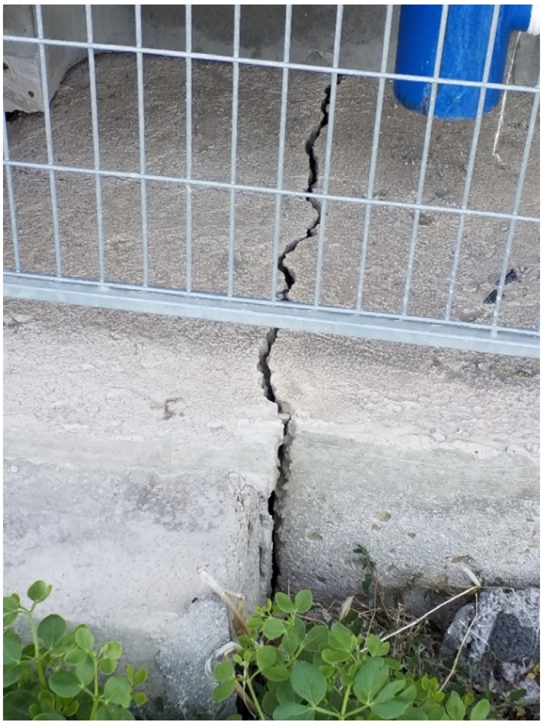

(c) (d)

Figure S3 | Monuments corresponding to GNSS stations 23 [panels (a) and (b)] and 28 [(c) and (d)]. The monument for station 23 has been moved horizontally due to water operations and the monument for station 28 had its base broken due to very local instability problems. Both monument problems result in the anomalous horizontal displacements at the SW part of the deformation area shown in Figure 3 of the main text. Photographs by authors.

Advanced Differential Synthetics Aperture Radar Interferometry (A-DInSAR)

The A-DInSAR technique allowed us to obtain deformation maps of the Alto Guadalentin Basin in line-of-sight (LOS) for the same time period as the GNSS campaigns. Due to the data availability we selected the C-Band Sentinel-1A satellite and used a Single-Look-Complex (SLC) image every two orbits, where available (see Table S2 for the acquisition dates of each orbit).

Using SUBSIDENCE-GUI software (Blanco-Sánchez et al., 2008), interferogram pairs were generated (see Table S3 and Table S4). For the ascending orbit a bigger set of interferograms were necessary due a greater atmospheric phase screen found in this geometry. For topographic phase removal a 5-meter DEM were used generated using Instituto Geográfico Nacional (IGN) MDT05 data.

Linear velocity and time series were calculated using Coherent Pixel Technique (Blanco-Sánchez et al., 2008). A mask of the zone were applied to reduce the computation time of the processing. Figure S4 shows the pixel's triangulations over the area, needed to calculate the linear velocity.

Figure S5 shows the calculated time-series of different points showing different patterns (stable points, subsidence and uplift).

Ascending orbit		Descending orbit	
20151112	20160721	20151212	20160727
20151206	20160814	20151224	**20160820**
20151230	20160907	20160117	20160913
20160123	20161001	20160210	20161007
20160216	20161025	20160305	20161031
20160311	20161106	20160329	20161124
20160404	20161130	20160422	20161218
20160428	20161224	20160516	20170111
20160510	20170105	20160609	20170204*
**20160603**	20170129	20160703	20170228
20160627	20170222		

Table S2. Acquisition dates of the SLC images used in this study. Bold numbers denote master images selected for registration. Marked with asterisk are those that were not used due to coregistration problems.

Ascending			
SLC1 – SLC2	SLC1 – SLC2	SLC1 – SLC2	SLC1 – SLC2
20151112 - 20151206	20160216 - 20160814	20160510 - 20160627	20160907 - 20161106
20151112 - 20151230	20160216 - 20160907	20160510 - 20160721	20160907 - 20161130
20151112 - 20160123	20160216 - 20161025	20160510 - 20160814	20160907 - 20161224
20151112 - 20160216	20160216 - 20161106	20160510 - 20161025	20160907 - 20170105
20151112 - 20160721	20160311 - 20160404	20160510 - 20161106	20160907 - 20170129
20151206 - 20151230	20160311 - 20160428	20160603 - 20160627	20160907 - 20170222
20151206 - 20160123	20160311 - 20160510	20160603 - 20160721	20161001 - 20161025
20151206 - 20160216	20160311 - 20160603	20160603 - 20160814	20161001 - 20161106
20151206 - 20160311	20160311 - 20160814	20160603 - 20160907	20161001 - 20161130
20151206 - 20160404	20160311 - 20160907	20160603 - 20161001	20161001 - 20161224
20151206 - 20160428	20160311 - 20161001	20160603 - 20161025	20161001 - 20170105
20151206 - 20160603	20160311 - 20161025	20160603 - 20161224	20161001 - 20170129
20151206 - 20160814	20160311 - 20161224	20160603 - 20170105	20161001 - 20170222
20151206 - 20160907	20160311 - 20170105	20160603 - 20170129	20161025 - 20161106
20151230 - 20160123	20160404 - 20160428	20160603 - 20170222	20161025 - 20161130
20151230 - 20160216	20160404 - 20160510	20160627 - 20160721	20161025 - 20161224
20151230 - 20160311	20160404 - 20160603	20160627 - 20160814	20161025 - 20170105
20151230 - 20160404	20160404 - 20160627	20160627 - 20160907	20161025 - 20170129
20151230 - 20160428	20160404 - 20160814	20160627 - 20161001	20161106 - 20161130
20151230 - 20160510	20160404 - 20160907	20160627 - 20161106	20161106 - 20161224
20151230 - 20160603	20160404 - 20161025	20160721 - 20160814	20161106 - 20170105
20151230 - 20160814	20160404 - 20161106	20160721 - 20160907	20161106 - 20170129
20151230 - 20160907	20160404 - 20170105	20160721 - 20161001	20161130 - 20161224
20151230 - 20161025	20160404 - 20170129	20160721 - 20161025	20161130 - 20170105
20160123 - 20160216	20160428 - 20160510	20160721 - 20161130	20161130 - 20170129
20160123 - 20160311	20160428 - 20160603	20160721 - 20161224	20161130 - 20170222
20160123 - 20160404	20160428 - 20160627	20160721 - 20170222	20161224 - 20170105
20160123 - 20160428	20160428 - 20160721	20160814 - 20160907	20161224 - 20170129
20160123 - 20160721	20160428 - 20160814	20160814 - 20161001	20161224 - 20170222
20160123 - 20161001	20160428 - 20160907	20160814 - 20161025	20170105 - 20170129
20160216 - 20160311	20160428 - 20161025	20160814 - 20161106	20170105 - 20170222
20160216 - 20160404	20160428 - 20161106	20160814 - 20170105	20170129 - 20170222
20160216 - 20160428	20160428 - 20170105	20160814 - 20170129	
20160216 - 20160510	20160428 - 20170129	20160907 - 20161001	
20160216 - 20160603	20160510 - 20160603	20160907 - 20161025	
Total: 137 Interferograms			

Table S3. Interferogram pairs obtained combining ascending radar images used in the DInSAR processing. All pairs were selected minimizing temporal or spatial baselines, avoiding those with high temporal and spatial baselines.

Descending	
**SLC1 – SLC2**	**SLC1 – SLC2**
20151212 - 20151224	20160609 - 20160703
20151212 - 20160117	20160609 - 20160727
20151212 - 20160210	20160609 - 20160913
20151224 - 20160117	20160703 - 20160727
20151224 - 20160210	20160703 - 20160820
20151224 - 20160305	20160703 - 20161031
20151224 - 20160422	20160703 - 20170111
20160117 - 20160210	20160727 - 20160820
20160117 - 20160305	20160727 - 20160913
20160117 - 20160727	20160820 - 20160913
20160210 - 20160305	20160820 - 20161007
20160210 - 20160329	20160820 - 20161218
20160210 - 20160422	20160913 - 20161007
20160210 - 20160820	20160913 - 20161031
20160305 - 20160329	20160913 - 20170228
20160305 - 20160422	20161007 - 20161031
20160329 - 20160422	20161007 - 20161124
20160329 - 20160516	20161007 - 20170228
20160329 - 20160913	20161031 - 20161218
20160422 - 20160516	20161031 - 20170111
20160422 - 20160609	20161124 - 20161218
20160516 - 20160609	20161124 - 20170111
20160516 - 20160703	20161124 - 20170228
20160516 - 20160913	20161218 - 20170111
Total: 48 Interferograms	

Table S4. Interferogram pairs obtained combining descending radar images used in the DInSAR processing. All pairs were selected minimizing temporal or spatial baselines, avoiding those with high temporal and spatial baselines.

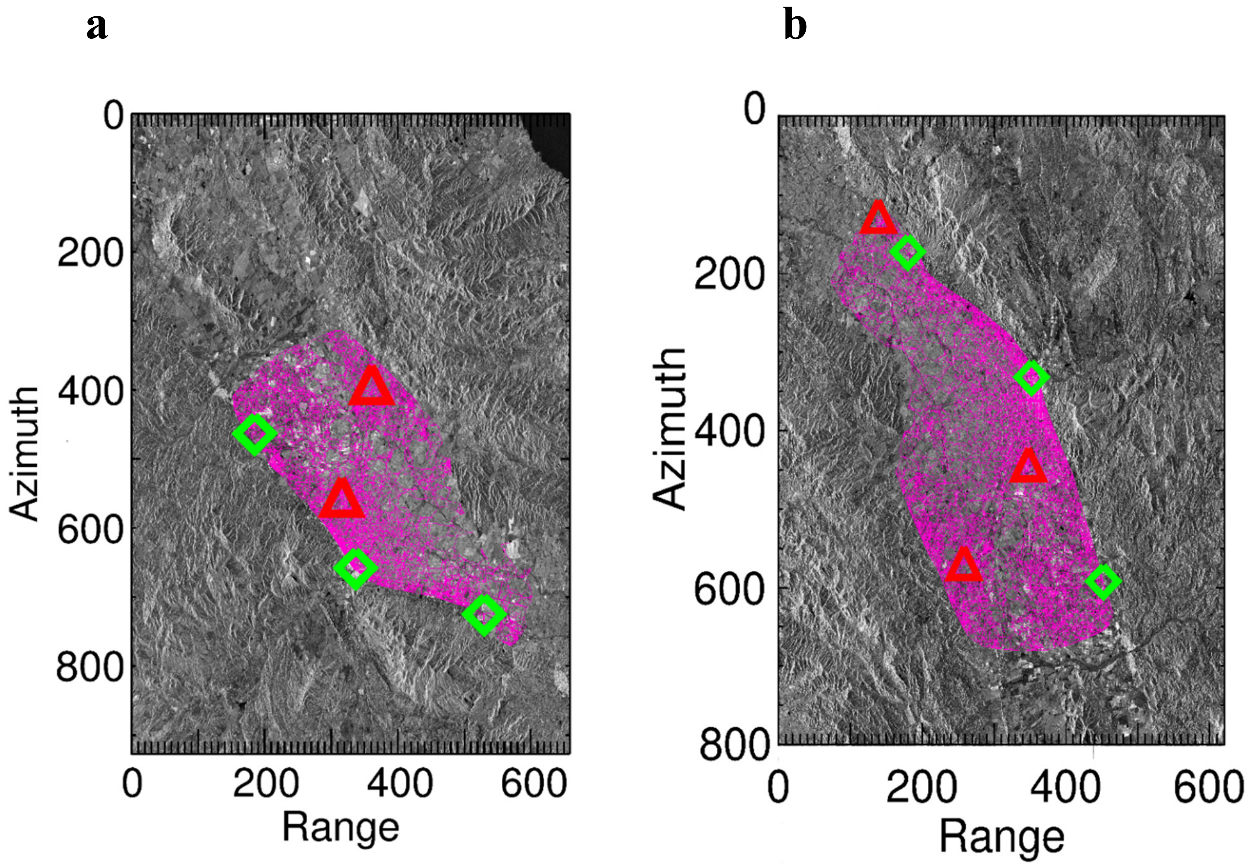

Figure S4 | Pixel triangulations over amplitude image maps for both geometries. (a) ascending and **(b)** descending orbit. CPT performs the integration of velocities using the Conjugate Gradient Method (CGM), an iterative and efficient method used for solving large systems of linear equations. This method requires points (seeds) with known velocity (green diamonds) and DEM error (red triangles) and performs a Delaunay triangulation between pixels (plotted in pink) to estimate the motion of the pixel. Stable points outside the deformation area has been used as velocity seeds and flat zones with good reflectivity as DEM error seeds. Connection graphs have been generated using SUBSIDENCE-GUI software (Blanco-Sánchez et al., 2008) and ESA Sentinel-1 toolbox (snap, step.esa.int/main/toolboxes/snap/).

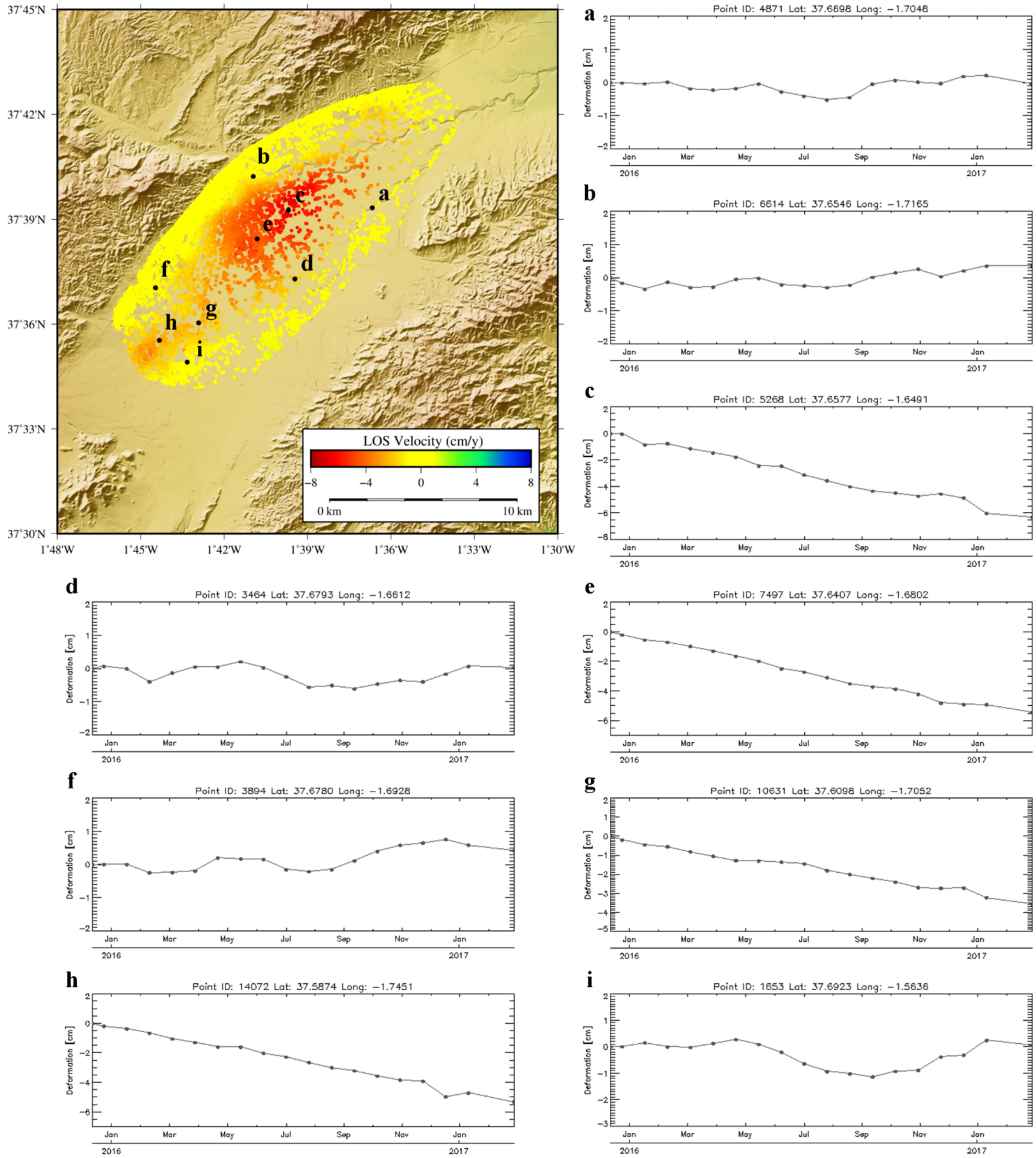

Figure S5 | A-DInSAR Time series for selected pixels in the deformation area. Pixels **a**, **b**, **d, f** and **i** show the measured signal in stable areas, which provides an indication of the noise/error level. Results are in agreement with the numerical values obtained from Table S6 and described in the main text (about ± 0.7 for LOS). The remainder of the pixels (**c**, **e**, **g** and **h**) show clear deformation time series with a very linear behavior. GMT software (Wessel et al., 2013) has been used to generate the map of the area of interest; times series has been generated using SUBSIDENCE-GUI software (Blanco-Sánchez et al., 2008)

Comparison of GNSS and A-DInSAR results

Since the A-DInSAR technique provide values of the velocity in LOS geometry, it cannot be compared directly with GNSS values. Having two geometries, East-West and Up-Down decomposition is possible, but still no information for the North-South component is available (see Methods section).

To compare both techniques, A-DInSAR and GNSS, we used two different approaches. First, we consider the E-W and vertical components using only those points which had a coherent pixel within 100 meters in both geometries and comparing the obtained values with the closest GNSS station. Results are shown on Table S5, it shows a difference of about 0.7 cm in the vertical component and 1.0 cm in the E-W.

Secondly we compare the LOS projected value of the GNSS velocity of each station with the A-DInSAR closest point. It is important to note that some of the GNSS stations are in low-coherence zones and do not have any close pixels, so for those points the results may be less accurate. Results shows good agreement between the two techniques, showing differences of about 0.7 cm as shown in the Table S6.

Table S5. Mean displacement rates over the studied period for GNSS and A-DInSAR (LOS and computed Up-Down and E-W components) techniques given in cm/year.

GNSS			A-DInSAR						
			Ascending Orbit		Descending Orbit		LOS Decomposition		
Station Number	Up-Down rate	E-W rate	D1 (m)	LOS rate	D2 (m)	LOS rate	D3 (m)	Up-Down rate	E-W rate
**05**	-8,3	-0,1	45	-6,7	33	-6,3	30	-9.0	1.3
**06**	-1,5	0,2	13	-1,2	41	-0,7	35	-1.4	0.0
**07**	-9.0	0,5	42	-7,0	84	-6,8	70	-9.5	1.5
**08**	-8,2	0,9	29	-7,0	56	-5,7	33	-9.0	1.0
**10**	-7,1	0,5	66	-6,4	62	-5,5	29	-8.3	0.9
**18**	-5,8	0,4	9	-4,2	22	-3,6	30	-5.4	0.5
**19**	-6,2	0,5	88	-4,8	64	-4,1	89	-6.1	0.6
**23**	-0,8	2,5	55	-1,0	63	-0,5	4	-1.0	-0.2
**24**	-0,6	0,8	37	-0,5	44	-0,5	8	-0.7	0.1
**27**	-4,1	0,4	28	-2,1	90	-1,9	58	-2.6	0.1

D1 (in m) denotes the distance of the closest coherent pixel in the ascending orbit results to the corresponding GNSS station. **D2** denotes the same as **D1** for descending orbit. **D3** denotes the distance between both pixels, ascending and descending, for the same GNSS station.

Table S6. Mean displacement rates over the studied period for GNSS (directly and projected LOS in ascending and descending orbits) and A-DInSAR (ascending and descending LOS) techniques given in cm/year.

GNSS						A-DInSAR			
						Ascending Orbit		Descending orbit	
Station	Up-Down rate	E-W rate	N-S rate	Ascending LOS	Descending LOS	D1	LOS	D2	LOS
01	-2,6	0,3	-0.7	-2.2	-1.8	31	-1,0	146	-1,5
02	-1,2	-0,2	-0.4	-0.8	-1.0	122	-1,2	27	-1,5
03	-0,7	-0,3	0.3	-0.4	-0.8	272	-0,5	324	-0,6
04	-1,3	-0,3	0.7	-1.0	-1.3	85	-0,4	124	-1,7
05	-8,3	-0,1	-0.4	-6.7	-6.6	45	-6,7	33	-6,3
06	-1,5	0,2	-0.6	-1.3	-1.0	13	-1,2	41	-0,7
07	-9.0	0,5	-1.1	-7.5	-6.7	42	-7,0	84	-6,8
08	-8,2	0,9	-1.1	-7.1	-5.8	29	-7,0	56	-5,7
09	-7,7	-0,6	0.4	-6.0	-6.5	270	-6,8	450	-6,1
10	-7,1	0,5	-0.1	-6.1	-5.3	66	-6,4	62	-5,5
11	-3.0	-0,4	0.2	-2.2	-2.7	157	-1,8	34	-2,2
12	-6,7	-0,4	0.0	-5.3	-5.5	115	-6,0	65	-5,3
13	-0,2	-0,6	0.6	0.1	-0.6	231	0,2	232	-1,0
14	0,4	-0,4	-1.1	0.7	0.2	232	0,5	150	-0,7
15	-2,2	-0,2	0.5	-1.7	-1.9	813	-1,1	721	-0,9
16	-3,6	-0,1	0.5	-3.0	-2.9	187	-2,7	157	-3,9
17	-1,7	-0,2	-0.9	-1.2	-1.4	125	-1,0	129	-0,7
18	-5,8	0,4	-0.6	-4.9	-4.3	9	-4,2	22	-3,6
19	-6,2	0,5	0.1	-5.3	-4.6	88	-4,8	64	-4,1
20	-2,7	-1,0	0.2	-1.6	-2.8	226	-2,1	156	-1,9
21	-0,7	-0,3	0.1	-0.4	-0.8	261	0,1	393	-0,5
22	-1,4	0,0	0.0	-1.2	-1.1	150	-0,2	157	-0,3
23	-0,8	2,5	0.1	-2.1	0.9	55	-1,0	63	-0,5
24	-0,6	0,8	-0.1	-0.9	0.0	37	-0,5	44	-0,5
25	-0,8	0,3	-0.4	-0.8	-0.4	205	-1,7	98	-2,3
26	-4,1	0,2	-0.4	-3.4	-3.1	165	-2,7	70	-2,7
27	-4,1	0,4	-0.4	-3.6	-2.9	28	-2,1	90	-1,9
28	-4	0,5	-2.2	-3.3	-2.6	240	-2,3	150	-2,4
29	-2,6	-0,4	0.4	-2.0	-2.3	77	-2,8	177	-2,7
30	-3,8	-0,1	-0.1	-3.1	-3.0	50	-2,0	130	-2,2
31	-1,1	0,3	-0.2	-1.0	-0.7	100	-2,6	62	-1,6
32	-2,2	-0,1	0.4	-1.8	-1.8	216	-3,5	102	-2,9
33	-5,3	-1,2	0.6	-3.7	-5.0	228	-4,6	23	-5,4

D1 (in m) denotes the distance of the closest coherent pixel in the ascending orbit results to the corresponding GNSS station. **D2** denotes the same as **D1** for descending orbit.

Inverse Modeling Results

As a complement to the inversion results shown in the main text, we describe here a more extended study. We employ ten different data sets of surface displacement (denoted as Cases) covering the period November 2015 to February 2017, and we carry out the inversion using the described forward model and inversion methodology in the Introduction and Methods section.

The Cases are the following:

(A) LOS A-DInSAR results obtained for descending orbit images, assuming 100% as vertical displacement.
(B) LOS A-DInSAR results obtained for ascending orbit images, assuming 100% as vertical displacement.
(C) Up-Down component obtained from the A-DInSAR, combining ascending and descending orbit images results.
(D) Purely LOS A-DInSAR results obtained for descending orbit images.
(E) Purely LOS A-DInSAR results obtained for ascending orbit images.
(F) Purely LOS A-DInSAR results obtained for ascending and descending orbit images.
(G) Up-Down and E-W A-DInSAR results obtained by combining ascending and descending orbit images results.
(H) 3D displacements determined using the GNSS surveys results.
(I) Purely LOS A-DInSAR results obtained for ascending and descending orbit images together with the 3D displacements determined using the GNSS surveys results.
(J) Up-Down and E-W A-DInSAR results obtained by combining ascending and descending orbit images, together with 3D displacements determined using the GNSS surveys results.

Cases **A**-**C** are one-dimensional (1D), **D**-**G** are 2D (either indirectly by combining Up-Down and E-W in any of the two measured LOS, or directly supplying the values for both displacement components separately), **H** is a purely 3D dataset, and **I** and **J** are a combination of 2D and 3D data (2D+3D data).

A very important difference between the purely GNSS 3D data and the rest of these cases is the number of displacement rate data points. For the 3D Case (**H**) data set we have just 108 displacement rates, while for the remainder we have thousands of measurements (see Table S7).

We invert each case and estimate the volume changes of the water table (volume and geometry) assuming a given pressure change value. Moreover, based on hydrogeological observations, we impose the criteria that sources are shallower than one kilometer. A summary of the results is provided in Table S7 and Figures S6 and S7. In these figures, the blue colors indicate negative pressure values, while white colors indicate positive pressure changes cells.

Inversion results include the volume and geometry of the active part of the aquifer which produced the measured displacements. Here this is quantified by the intensity, equal to the product of volume by pressure change, as it is impossible to determine both quantities separately. If we increase pressure, we decrease volume and vice versa. In order to determine a general geometry, we have constrained the value of pressure change (Camacho et al., 2011; Cannavò et al., 2015). After a trial analysis, we consider a pressure value of -3 MPa, selecting the value that gives us a source geometry most consistent with the characteristics of the Lorca aquifer.

Note that the inversion results obtained from these data sets can be organized into two subsets: (i) Cases **A**-**C** (1D, vertical displacement) and (ii) Cases **D**-**J** (2D and 2D+3D). Both sets of results are internally consistent, with respective scattering on the order of 9 and 6% respectively (Table S7).

The results of (i) are, on average, ~26% greater in intensity/volume than those of (ii), indicating that using only one component of the displacement field and assuming that displacement are only vertical significantly overestimates the volume of water extracted during the study period (on the order of tens of hm^3). This can have an important effect in predictions of future volume variations and surface displacements.

The results for group (i) show the largest discrepancies between Case **C** and the other two cases (**A** and **B**). For Case **C** we obtain an intensity value ~20% greater than the other ones. This data set has been obtained from combining LOS results from ascending and descending orbits and they suffer from the small systematic errors in the methodology. These include the non-application of the minor correction due to the squint angle as well as possible errors coming from interpolation required because the pixels identified for ascending and descending satellite orbits are not identical, combined with the small amplitude of the E-W displacements for most of the pixels. This problem is reflected in two aspects, the poorer misfit values and the appearance of additional sources at the edges of the study area that try to adjust these errors (see Figure S7). As a result, it clearly is more appropriate, as shown in previous works (Fokker et al., 2016), to mitigate this problem by using the ascending and/or descending LOS directly in the inversion procedure. In future work we will investigate the results if we estimate the vertical and E-W components using a more precise methodology, e.g. MSBAS (Samsonov et al., 2012).

As a result, we do not consider the results for Case **C** and use only those results from Cases **A** and **B**. We obtain very consistent results (see Table S7), with a dispersion of ~4% for group (i) and a mean intensity value of 40 $MPa \cdot km^3$.

For the group (ii) results, the data set for the vertical and E-W components determined using the combination of ascending and descending LOS (Cases **G** and **J**) (Table S7) again provides worst results (higher intensities, more additional sources and poorer misfit values, Figure S7 and Table S7). Case **H** (only GNSS determined displacements) also provides higher misfits (Table S7) and poorer adjusted geometries (Figure S7h), probably a result of using data with several important limitations. The first is the reduced number of data points available in relation to the number of unknown parameters (approximately one hundred versus several thousands, see Table S7 for details). As previously mentioned, in cases like this, a large number of measurement points are needed to reliably estimate the distribution of reservoir volume changes (Vasco et al., 2002). Also, their limited number produces a poorer spatial distribution than A-DInSAR data and does not cover the entire deformed area (see Figure 5). And finally, as described previously, in this reduced data set we have some anomalous results associated to very local effects (Figure 3b and Figure S3 in this Supplementary Information). To establish a denser and more extended GNSS network requires additional cost and time for field observation.

<table>
<tr><th colspan="8">Table S7. Numerical summary of the inversion results obtained for the ten cases.</th></tr>
<tr><th>CASE</th><th>Intensity (MPa×Km^3)</th><th>Misfit (cm)</th><th>Mean Model Intensity (MPa×Km^3)</th><th>Pres. (MPa)</th><th>Vol. (Km^3)</th><th>Displacement components considered</th><th>Number of data used</th></tr>
<tr><td>A</td><td>-41</td><td>0.36</td><td rowspan="3">-42.7 ± 3.8 (9%)
[-40.0 ± 1.4 (4%)]</td><td rowspan="3">-3</td><td rowspan="3">14.2
[13.3]</td><td rowspan="3">1D</td><td>1505</td></tr>
<tr><td>B</td><td>-39</td><td>0.31</td><td>1203</td></tr>
<tr><td>C</td><td>-48</td><td>0.19</td><td>1572</td></tr>
<tr><td>D</td><td>-32</td><td>0.30</td><td rowspan="7">-33.9 ± 1.9 (6%)
[-32.5 ± 1.1 (3%)]</td><td rowspan="7">-3</td><td rowspan="7">11.3
[10.8]</td><td rowspan="4">2D</td><td>1505</td></tr>
<tr><td>E</td><td>-31</td><td>0.28</td><td>1203</td></tr>
<tr><td>F</td><td>-33</td><td>0.32</td><td>2708</td></tr>
<tr><td>G</td><td>-37</td><td>0.45</td><td>3144</td></tr>
<tr><td>H</td><td>-34</td><td>0.94</td><td>3D</td><td>108</td></tr>
<tr><td>I</td><td>-34</td><td>0.43</td><td rowspan="2">2D+3D</td><td>2816</td></tr>
<tr><td>J</td><td>-36</td><td>0.63</td><td>3252</td></tr>
<tr><td colspan="8">Data are grouped into two types: 1D data (Cases A-C), 2D and 2D +3D (Cases D-J). 2D data come from A-DInSAR results. 2D+3D data sets combine data obtained from the A-DInSAR study with those obtained from GNSS observation campaigns. For each of these two sets, which give rise to very similar results, mean values of intensity and values of volume variation in the aquifer, as a function of the pressure variation assumed, are given. Blue color figures denote average values determined not considering Cases C, and Cases G, H and J respectively. See text for more details.</td></tr>
</table>

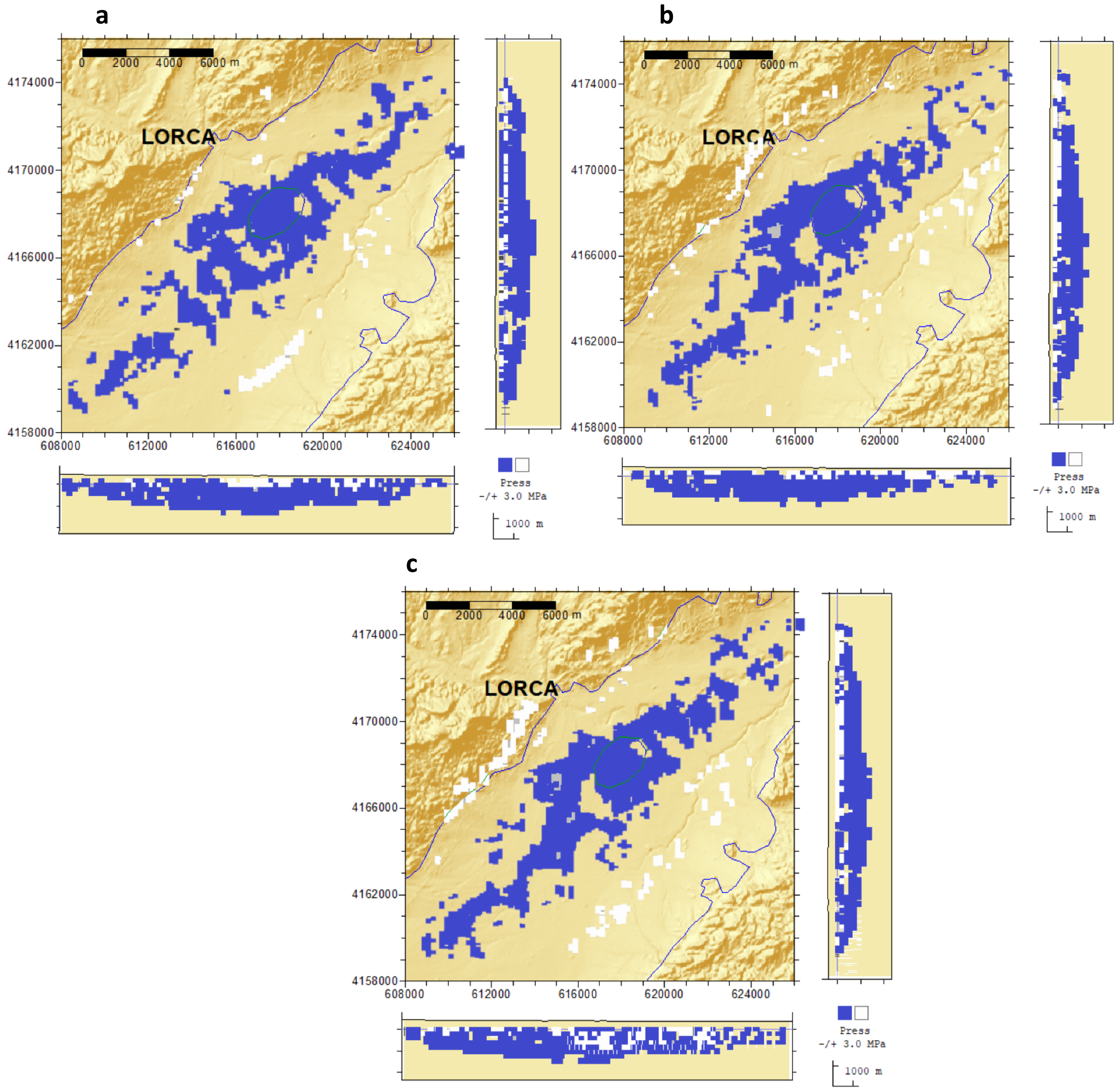

Figure S6 | Representation of the inversion results obtained for the 1D considered data sets. **(a)** Obtained source for Case **A**; **(b)** for Case **B**, and **(c)** for Case **C**. Blue color indicates negative pressure value cells, produced by water extraction. White color indicates positive pressure change cells. These positive pressure sources adjust the errors and the effects of other deformation sources, different from water extraction (e.g., of tectonic origin). This figure was created using Surfer 8.02 Surface Mapping System (www.goldensoftware.com/products/surfer) and Paint, Microsoft Windows 10.

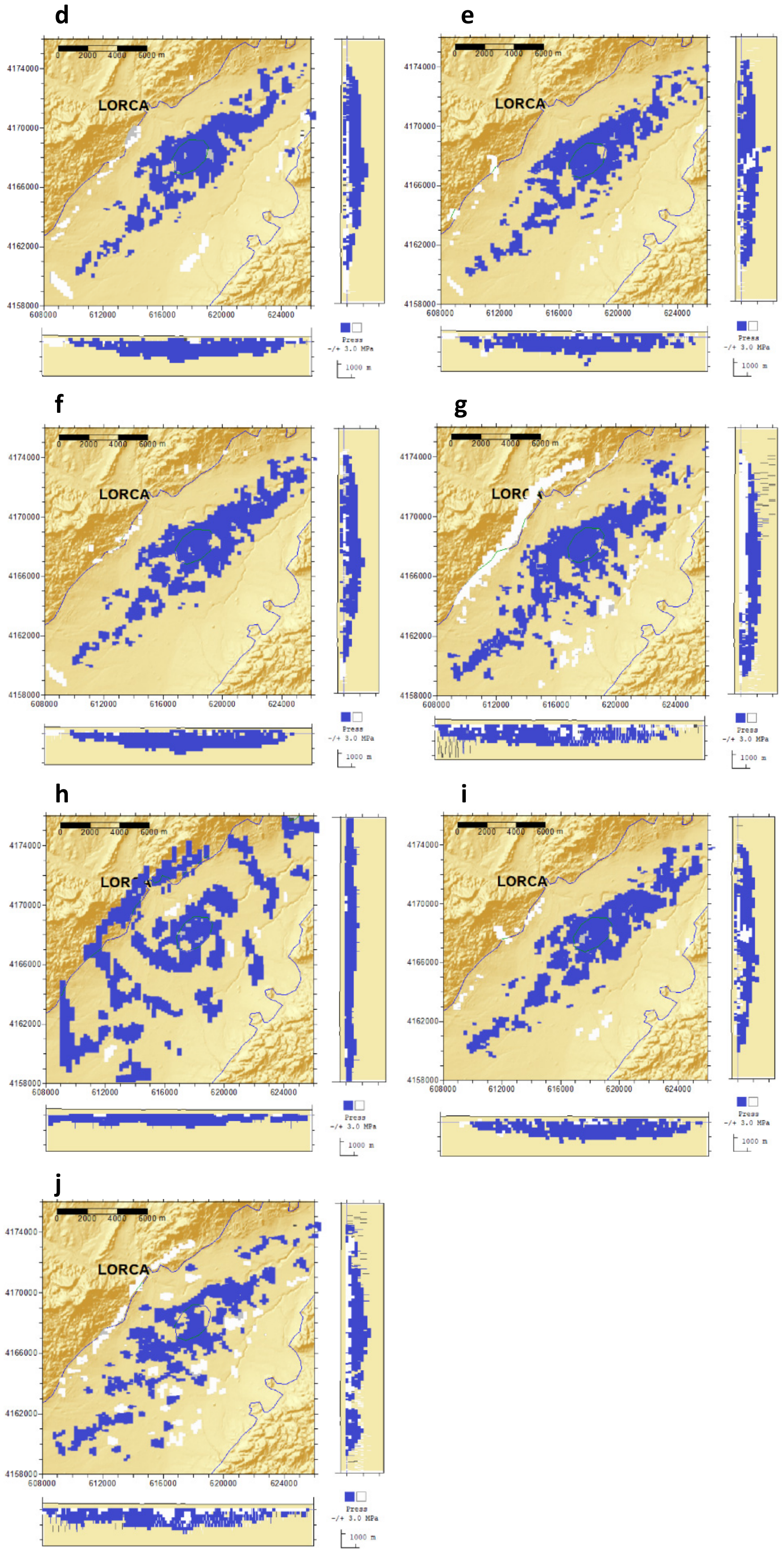

Figure S7 | Representation of the inversion results obtained for the 2D, 3D and 2D+3D data sets. (d) Results for Case **D**, **(e)** for Case **E**; **(f)** for Case **F**, **(g)** for Case **G**, **(i)** for Case **I**, and **(j)** for Case **J**. Blue color indicates negative pressure value cells, produced by water extraction. White color indicates positive pressure change cells. These positive pressure sources adjust the errors and the effects of others deformation sources different from water extraction. This figure was created using Surfer 8.02 Surface Mapping System (www.goldensoftware.com/products/surfer) and Paint, Microsoft Windows 10.

Synthetic test case

To compare our study to one in which the horizontal displacements are more significant with respect to the vertical than for Lorca, we consider a synthetic test case. For an area similar to the Lorca study area, we simulate displacements due to a buried low pressure body. It is constituted by an ellipsoidal disk (Figure S8a) with semi-axes a=2000 m and b=4000 m, thickness 1000 m, depth of the top 200 m, oriented with azimuth E 50ºN, and pressure contrast of -2 MPa.

For this simulated active body, we calculate the deformation effect on a set of 3127 observation points (see Figure S9) with a gridded distribution within a circular area (radius = 7000 m) above the ellipsoidal disk. The deformation values (upward u_z, easting u_x and northing u_y components) due to the homogeneously pressurized body are determined according the improved Geertsma method (Geertsma and Van Opstal, 1973; Brown et al., 2014). We divided the complete pressurized body into 2509 small rectangular prisms. Figure S8 shows the pattern of the u_z, u_x, and u_y deformation values.

To simulate InSAR data, we also estimate the displacement on the surface points measured along the radar line of sight (LOS) corresponding to the direction of the satellite. Then, the modeled changes u_{LOSi} for the n data points can be written as:

$$u_{LOSi} = u_{zi} \cdot \cos\beta + u_{xi} \cdot \sin\beta \cdot \cos\alpha - u_{yi} \cdot \sin\beta \cdot \sin\alpha;\ i=1,\ldots,n$$

where α, β are the direction angles (azimuth and incidence) for the antenna pointing direction and u_{xi}, u_{yi}, u_{zi} are the components of deformation along the axes. It is applied to both he ascending and descending orbits. Figure S9d,e shows the patterns of the LOS displacement values for both orbit cases.

For these different data sets, we apply the described inverse approach (see main text, Methods section) to model the causative body. Figure S8b shows the adjusted causative structure obtained as an aggregation of 8600 cells for the case of considering directly the computed three component deformation data. We observe in the geometrical pattern of the solution structure, apart from a general good fit, a clear rounding of the sharp (deepest) geometrical details. This is due to the smoothness constraints, included here to obtain a unique solution.

The inversion approach is carried out for several combinations of data sets, simulating we have observed ascending and descending LOS:

- **(SA)** LOS A-DInSAR results obtained for descending orbit images, considering 100% as vertical displacement.
- **(SB)** LOS A-DInSAR results obtained for ascending orbit images, considering 100% as vertical displacement.
- **(SC)** Purely LOS A-DInSAR results obtained for descending orbit images.
- **(SD)** Purely LOS A-DInSAR results obtained for ascending orbit images.
- **(SE)** LOS A-DInSAR results obtained for ascending and descending orbit images.

Table S8 shows a comparative study about the resulting model size for the several cases.

a)

b)

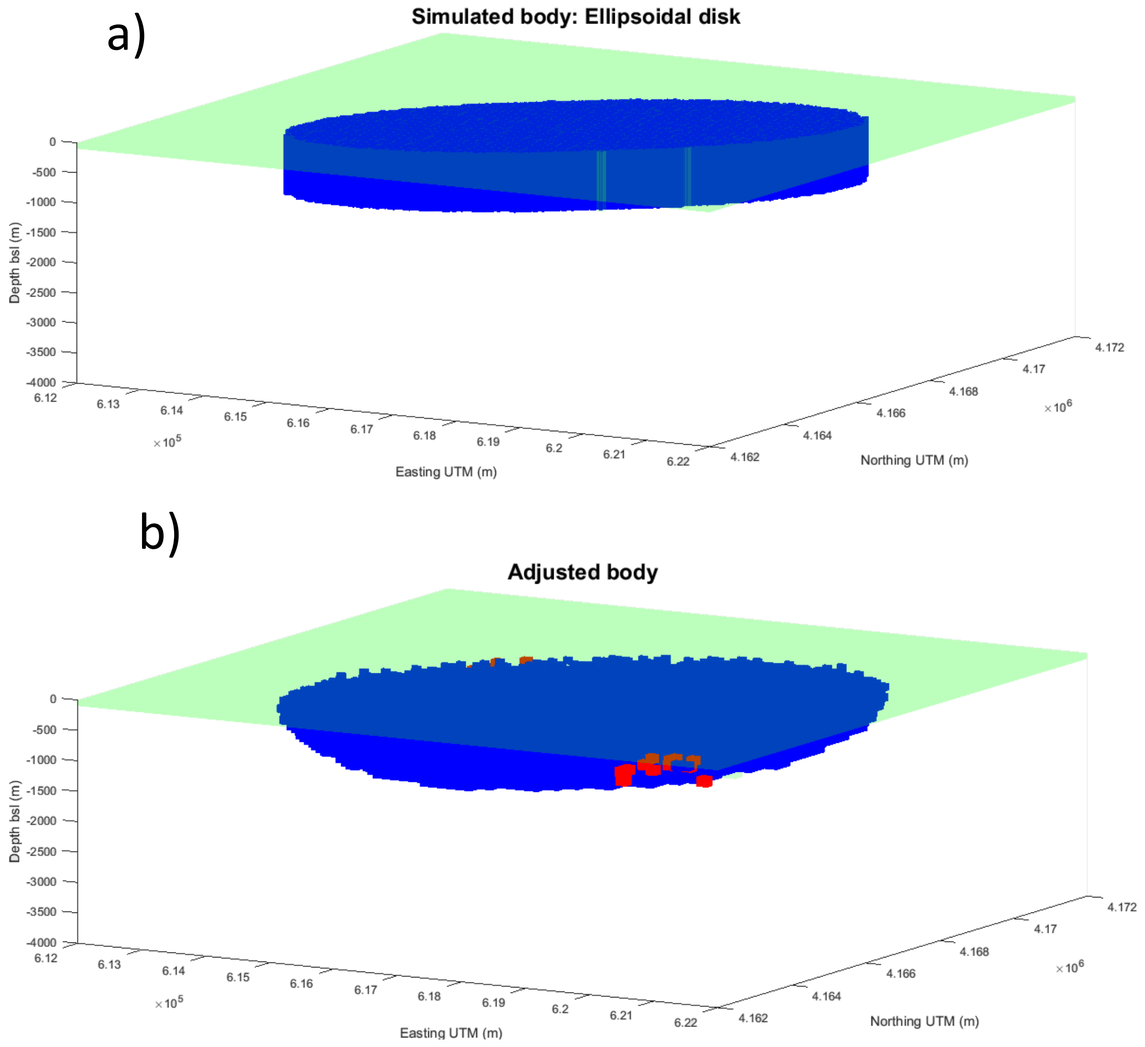

Figure S8 | Synthetic test case. (**a**) Simulated horizontal ellipsoidal disk for homogeneous low pressure Δp= -2 MPa. (**b**) Adjusted causative structure obtained as aggregation of 8600 cells inverted from the data set composed by the computed 3D displacement data.

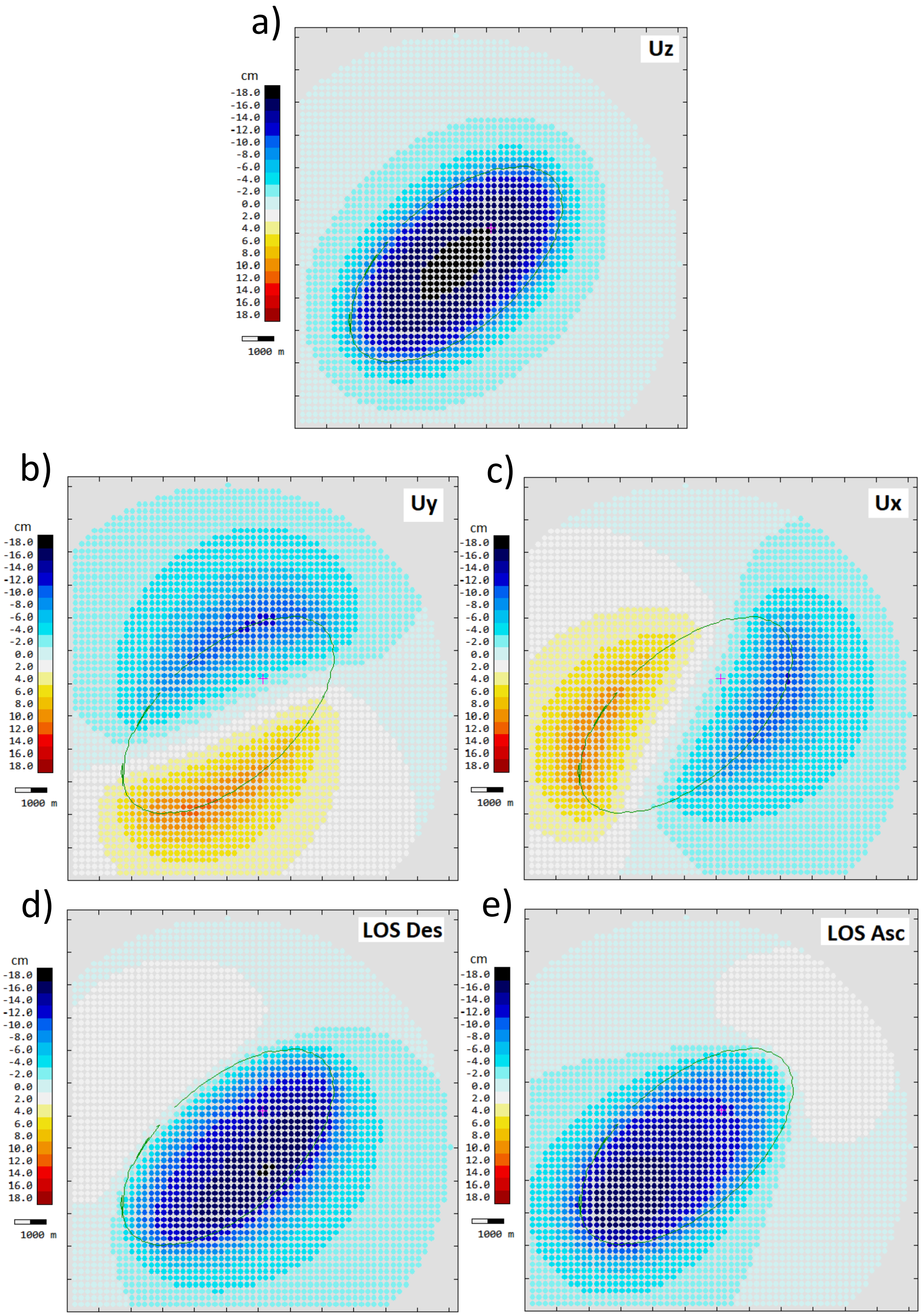

Figure S9 | Simulated displacements values due to the elliptical disk on a gridded distribution of data points. (**a**) Vertical component; (**b**) E-W component; (**c**) N-S component; (**d**) ascending LOS ascending values; (e) descending LOS values.

Table S8. Numerical summary of the inversion results obtained for the five synthetic cases.						
**CASE**	**Intensity (MPa×Km^3)**	**Misfit (cm)**	**Pressure (MPa)**	**Vol. (Km^3)**	**Displacement components**	**Discrepancies with 2D results**
**SA**	-68	0.42	-2	34	1D	28%
**SB**	-60	0.37		30		13%
**SD**	-53	0.31		26.5	2D	0%
**SE**	-53	0.30				
**SF**	-53	0.30				

Supplementary References

Blanco-Sánchez, P., Mallorquí, J. J., Duque, S. & Monells, D., 2008. The Coherent Pixels Technique (CPT): An Advanced DInSAR Technique for Nonlinear Deformation Monitoring. Pure Appl. Geophys. 165, 1167–1193.

Bonì, R., Herrera, G., Meisina, C., Notti, D., Béjar-Pizarro, M., Zucca, F., González, P.J., Palano, M., Tomás, R., Fernández, J., Fernández-Merodo, J.A., Mulas, J., Aragón, R., Guardiola-Albert, C., Mora, O., 2015. Twenty-year advanced DInSAR analysis of severe land subsidence: The Alto Guadalentín Basin (Spain) case study. Engineering Geology, 198, 40–52 doi:10.1016/j.enggeo.2015.08.014

Brown, N. J., Woods, A.W., Neufeld, J. A., Richardson, C. 2014. Constraining Surface Deformation Predictions Resulting from Coal Seam Gas Extraction. Record 2014/14. Geoscience Australia, Canberra. http://dx.doi.org/10.11636/Record.2014.044

Camacho, A.G., González, P.J., Fernández, J., Berrino, G., 2011. Simultaneous inversion of surface deformation and gravity changes by means of extended bodies with a free geometry: Application to deforming calderas. Journal of Geophysical Research, 116, B10401, doi: 10.1029/2010JB008165.

Cannavò, F., Camacho, A.G., González, P.J., Mattia, M., Puglisi, G., Fernández, J., 2015. Real Time Tracking of Magmatic Intrusions by means of Ground Deformation Modeling during Volcanic Crises. Scientific Reports, 5, 10970, doi: 10.1038/srep10970.

Fokker, P. A., Wassing, B. B. T., van Leijen, F. J., Hanssen, R. F., Nieuwland, D. A., 2016. Application of an ensemble smoother with multiple data assimilation to the Bergermeer gas field, using PS-InSAR. Geomech. Energy Environ. 5, 16–28.

Geertsma, J., Van Opstal, G., 1973. A numerical technique for predicting subsidence above compacting reservoirs based on the nucleus of strain concept, Verhandelingen Kon. Ned. Geol. Mijnbouwk, 28, 63-78.

González, P.J., Fernández, J., 2011. Drought-driven transient aquifer compaction imaged using multitemporal satellite radar interferometry. Geology, 39(6), 551–554; doi: 10.1130/G31900.1.

González, P.J.; Tiampo, K.F.; Palano, M.; Cannavó, F.; and Fernández, J., 2012. The 2011 Lorca earthquake slip distribution controlled by groundwater crustal unloading. Nature Geoscience, 5(11), 755-834. doi: 10.1038/NGEO1610.

Herring, T. A., King, R. W., Floyd, M. A. & McClusky, S. C., 2015. GAMIT Reference Manual. GPS Analysis at MIT GLOBK, Release 10.6. (Massachusetts Institute of Technology).

Samsonov, S., D'Oreye, N., 2012. Multidimensional time-series analysis of ground deformation from multiple InSAR data sets applied to Virunga Volcanic Province. Geophys. J. Int. 191, 1095–1108.

Vasco, D. W., Wicks, C., Karasaki, K., Marques, O., 2002. Geodetic imaging: reservoir monitoring using satellite interferometry. Geophys. J. Int. 149, 555–571.

Wessel, P.; Smith, W. H.; Scharroo, R.; Luis, J. F., Wobbe, F., 2013. Generic Mapping Tools: Improved version released. EOS Trans. AGU, (94), pp. 409–410. doi:10.1002/2013EO450001.
